# Practical utility of meropenem therapeutic drug monitoring: a systematic review of evidence for clinical application

**DOI:** 10.3389/fphar.2025.1725419

**Published:** 2025-12-11

**Authors:** Yi-Chang Zhao, Jia-Yi Liu, Jia-Kai Li, Huai-Yuan Liu, Zhi-Hua Sun, Bi-Kui Zhang, Miao Yan

**Affiliations:** 1 Department of Pharmacy, The Second Xiangya Hospital, Central South University, Changsha, Hunan, China; 2 International Research Center for Precision Medicine, Transformative Technology and Software Services, Changsha, Hunan, China; 3 School of Basic Medicine and Clinical Pharmacy at China Pharmaceutical University, Nanjing, Jiangsu, China

**Keywords:** therapeutic drug monitoring, meropenem, clinical outcomes, antibiotictherapy, meta-analysis

## Abstract

**Background and purpose:**

Therapeutic Drug Monitoring (TDM) is advocated to optimize antibiotic therapy, yet its efficacy remains debated. This systematic review and meta-analysis evaluates TDM’s impact on meropenem therapy, focusing on treatment efficacy, bacterial clearance, and changes in biomarkers (C-reactive protein (CRP) levels, procalcitonin (PCT) levels, white blood cell (WBC) count, neutrophil ratios).

**Methods:**

A systematic search was conducted across multiple databases, including PubMed, Embase, Web of Science, the Cochrane Library, and CNKI. Eligible studies comparing TDM with non-TDM approaches for meropenem were identified according to predefined inclusion and exclusion criteria. Pooled analyses were conducted using fixed-effects or random-effects models, selected based on the degree of heterogeneity among studies.

**Results:**

A total of nine studies comprising 834 patients met the inclusion criteria and were included in the analysis. The study populations primarily consisted of adult and elderly patients with moderate to severe infections, including respiratory tract, urinary tract, and bloodstream infections. TDM significantly improved treatment efficacy (RR = 1.19; 95% CI: 1.10–1.28) and bacterial clearance rates (RR = 1.29; 95% CI: 1.12–1.48). CRP levels decreased significantly (mean difference = 14.91; 95% CI: 2.79–27.02), while changes in PCT, WBC, and neutrophil ratios were non-significant. Adverse reactions showed a non-significant reduction (RR = 0.65; 95% CI: 0.38–1.11). Low heterogeneity across studies supports the robustness of these findings.

**Conclusion:**

TDM enhances treatment efficacy and bacterial clearance in meropenem therapy, but its impact on adverse reactions and certain biomarkers remains uncertain. Further research is needed to clarify TDM’s clinical utility and limitations.

## 1 Introduction

Meropenem is classified as a carbapenem-type β-lactam antibiotic, characterized by its broad-spectrum activity and low toxicity. It effectively targets a variety of microorganisms. However, for carbapenem-resistant Enterobacterales (CRE), which are resistant to carbapenems, alternative treatment options such as polymyxins, tigecycline, or newer β-lactam/β-lactamase inhibitor combinations (e.g., ceftazidime-avibactam) are typically required. Nonetheless, it remains a valuable and commonly prescribed option for treating severe infections and hospital-acquired infections in hospitalized patients when susceptibility is confirmed (Streit et al., 2016; Gutiérrez-Gutiérrez et al., 2017; Gonçalves-Pereira and Póvoa, 2011).

Given the increasing understanding of the connections between antimicrobial dosing and patient outcomes, there is a compelling argument for tailoring antimicrobial dosing to the specific needs of ill patients through the utilization of Therapeutic Drug Monitoring (TDM), which involves measuring drug concentrations in a patient’s blood to optimize dosing regimens and ensure therapeutic efficacy while minimizing toxicity (Tabah et al., 2015). TDM has been proven to be a powerful tool for achieving personalized therapy, aiding in optimizing exposure, which is associated with enhanced clinical outcomes (Muller et al., 2018). To illustrate, experts recommend routine TDM for aminoglycosides, beta-lactam antibiotics, linezolid, teicoplanin, vancomycin, and voriconazole in critically ill patients (Abdul-Aziz et al., 2020). Besides, previous research found that meropenem exhibited time-dependent and Ctrough/MIC >4 is a potential instrument to predict successful treatment with meropenem (Streit et al., 2016; Zhao et al., 2022).

In recent years, numerous clinical studies have explored the role of TDM in optimizing meropenem therapy, with mixed results. Some studies support TDM’s clinical benefits, while others do not. These studies provide crucial evidence for implementing meropenem TDM but have limitations. The number of patients in some studies was small, limiting the statistical ability to capture the effects of TDM. Specifically, only 25 patients were included in a study about the safety of patients with TDM (Schmid et al., 2023). Study designs lack diversity, often using observational designs instead of randomized trials, impacting result reliability. Some studies evaluated the pre-analytical stability of TDM through structured review (Mortensen et al., 2022), and others explored TDM in critically ill patients through retrospective cohort studies (Dräger et al., 2023). Despite guidelines recommending TDM with meropenem (Guilhaumou et al., 2019), its clinical implementation varies widely, with significant differences in strategies, frequency, and techniques among healthcare institutions. As a case in point, in critically ill or immunocompromised patients, studies have indicated the need for more aggressive pharmacodynamic targets (100% T > MIC) (Pea and Viale, 2009) and higher targets (4–6 × 100% T > MIC) (Wong et al., 2014). Additionally, most studies are conducted within specific geographic regions, which may not adequately represent the treatment outcomes for patients in other areas (Kühn et al., 2020; Schoenenberger-Arnaiz et al., 2020). In summary, more evidence is needed to fully assess the value and effectiveness of TDM in meropenem therapy.

Therefore, this study is dedicated to systematically assessing the utility of TDM in meropenem therapy, particularly its impact on therapeutic outcomes and safety, through a meta-analysis of the available evidence. Previous reviews, such as the one by Steffens et al. (2021), have demonstrated the benefits of TDM but did not specify the impact on key outcomes, including treatment efficacy, bacterial clearance rates, adverse reactions, C-reactive protein (CRP) levels, procalcitonin (PCT) levels, white blood cell (WBC) count, neutrophil ratios (Steffens et al., 2021). To remedy this deficit in the existing literature, our study aims to provide a comprehensive evaluation of the existing evidence and its clinical relevance. The evaluation of the actual utility of TDM in meropenem therapy will support precise dose and frequency adjustments clinically, reducing drug-related adverse reactions and enhancing therapeutic effects. Additionally, this systematic review will provide important references for developing future clinical guidelines and health policies to optimize meropenem drug therapy management, thereby improving overall healthcare quality and ensuring patient treatment safety.

## 2 Materials and methods

### 2.1 Search strategy

A literature search was conducted in five databases, including four English-language databases (PubMed, Web of Science, Embase, Cochrane Library) and one Chinese database (CNKI), with the search timeframe extending from the inception of each database to May 2024.

The search strategy employed both Medical Subject Headings (MeSH) and free-text terms related to the intervention, study population, and outcomes. The search focused on three main concepts: (1) “Meropenem,” (2) “Therapeutic Drug Monitoring” OR “TDM,” and (3) “Clinical Trial” OR “Randomized Controlled Trial.” Reference lists of all eligible studies, systematic reviews, and meta-analyses were also manually screened to identify additional relevant records. All retrieved references were imported into EndNote (Clarivate Analytics) for reference management. Duplicate and irrelevant entries were identified and removed to ensure accuracy and consistency in study selection. For studies published in Chinese, data extraction and translation were independently conducted by two bilingual researchers to minimize potential translation bias, and all extracted data were cross-checked and verified by a third reviewer. This approach ensured both linguistic accuracy and methodological reliability in handling non-English literature. A detailed description of the complete search strategy, including database-specific parameters and full search strings, is provided in Supplementary File 1.

### 2.2 Inclusion and exclusion criteria

The inclusion criteria for this study were meticulously established to ensure methodological rigor and relevance. We included all randomized controlled trials (RCTs) and clinical trials that assessed the therapeutic efficacy of meropenem using TDM, defined as the measurement of meropenem plasma concentrations to optimize dosing regimens and ensure target attainment (e.g., achieving pharmacokinetic/pharmacodynamic (PK/PD) targets such as %T > MIC), compared with traditional empirical treatments. These studies were required to incorporate meropenem within the treatment regimen and report on primary outcomes such as clinical efficacy, as well as secondary outcomes like safety outcomes (e.g., incidence of adverse reactions), microbiological clearance rates, and variations in some inflammatory markers such as CRP levels, WBC count, and PCT levels. Studies that reported only primary outcomes (treatment efficacy) were also included, even if safety or laboratory outcomes were not available. Similarly, studies that reported only laboratory markers were considered eligible if they also evaluated TDM and meropenem therapy. The exclusion criteria were strictly defined to refine the scope and quality of the evidence, excluding sources where the original text was unavailable, studies not focused on clinical treatment outcomes, review-type articles lacking original research data, and experimental studies that failed to integrate TDM. This study was conducted by the International Prospective Register of Systematic Reviews (PRISMA) guidelines (Sideri et al., 2018) and registered in PROSPERO, the International Prospective Register of Systematic Reviews, with the registration number CRD42024546358.

### 2.3 Outcomes selection

This study comprehensively evaluated several key indicators to measure the efficacy and safety of the treatment. The primary outcome measure was the treatment efficacy rate, which reflected the extent of improvement in patients’ symptoms and signs post-treatment. Treatment efficacy was defined as the proportion of patients who achieved clinical improvement or complete resolution of infection following meropenem treatment guided by TDM. Clinical improvement was primarily assessed by reductions in fever, improvements in respiratory status, and overall clinical recovery. Additionally, microbiological clearance, which is defined as the eradication of the causative pathogen from clinical specimens, was considered a key indicator of treatment success. Treatment efficacy was calculated using the relative risk (RR) of treatment success in patients who received TDM versus those who received non-TDM approaches. Specifically, the efficacy was measured by comparing the proportion of patients achieving treatment success (defined as resolution of infection or significant clinical improvement) in both groups. The RR is calculated as:

$$RR=\frac{\text{Risk}\;\text{of}\;\text{treatment}\;\text{success}\;\text{in}\;\text{the}\;\text{TDM}\;\text{group}}{\text{Risk}\;\text{of}\;\text{treatment}\;\text{success}\;\text{in}\;\text{the}\;\text{non}‐\text{TDM}\;\text{group}}$$

For each study, the risk of treatment success was determined by the number of patients with successful treatment outcomes divided by the total number of patients in each group. The pooled RR was derived from all included studies, with 95% confidence intervals (CIs) to assess the precision of the estimate. A statistically significant improvement in treatment efficacy was considered when the RR for the TDM group was greater than 1, indicating a higher likelihood of treatment success compared to the non-TDM group.

Additionally, we assessed the following indicators: bacterial clearance rate, representing the proportion of bacteria completely eradicated after treatment; changes in inflammatory markers such as CRP, WBC, and PCT levels, which are commonly used to assess the body’s inflammatory status and the severity of bacterial infections. We also evaluated the neutrophil ratio, indicating the proportion of neutrophils to other white blood cells, useful for assessing infection or inflammation levels; and the incidence of adverse reactions post-treatment, which helps evaluate the safety of the treatment.

### 2.4 Data extraction and evaluation

In this study, data from selected research articles that met our inclusion and exclusion criteria were organized and analyzed using Microsoft Excel. This process involved categorizing key data points such as literature characteristics (author, publication year), study design (type of research, sample size), intervention measures, basic patient information, and outcome measures. The methodological quality of the included studies was systematically evaluated using the Cochrane Risk of Bias Tool implemented in RevMan (Review Manager 5.4.1), which assesses potential sources of bias across multiple domains including random sequence generation, allocation concealment, blinding, incomplete outcome data, selective reporting, and other sources of bias (Higgins et al., 2011). The evaluation criteria included random sequence generation, allocation concealment, blinding of participants and personnel, completeness of outcome data, selective reporting, and other potential sources of bias. Two researchers independently performed data extraction and quality assessment to ensure accuracy and reliability, and any discrepancies were resolved through discussion or, when necessary, consultation with a third reviewer.

### 2.5 Efficacy and safety outcomes meta-analysis

In the methodology of this study, we delineated two groups for comparison: the control group comprised patients solely undergoing meropenem treatment (non-TDM group), whereas the intervention group included patients receiving meropenem alongside therapeutic drug monitoring (TDM group). Outcomes from both groups were systematically compared and evaluated. The primary endpoint was the clinical efficacy rate, which quantifies the proportion of patients exhibiting improvements in clinical symptoms and signs post-treatment. Secondary endpoints encompassed the microbiological clearance rate—the percentage of patients achieving total bacterial eradication following treatment—and changes in biomarkers indicative of inflammation and infection status, including CRP levels, WBC count, PCT levels, and neutrophil ratios. Safety assessments focused on the incidence of adverse reactions. Studies that reported only primary outcomes (treatment efficacy) were included, even if secondary outcomes were unavailable.

### 2.6 Heterogeneity and model selection

We performed meta-analyses using fixed-effects and random-effects models according to the extent of between-study heterogeneity. Heterogeneity was quantified with the I^2^ statistic (with values around 25%, 50%, and 75% interpreted as low, moderate, and high, respectively) and the Cochran Q test (p < 0.10 indicating statistical heterogeneity). A fixed-effects model was applied when clinical and methodological characteristics were comparable and heterogeneity was negligible to low (non-significant Q, I^2^ ≲ 50%), under the assumption of a common true effect. When heterogeneity was moderate to high (I^2^ > 50% and/or Q p < 0.10), or when meaningful clinical diversity (e.g., patient severity, infection site, dosing strategies) was anticipated, we used a random-effects model (DerSimonian–Laird), which assumes study-specific true effects distributed around an average effect. RR with CIs were pooled for dichotomous outcomes; mean differences (MDs) with 95% CIs were pooled for continuous outcomes. For statistical analysis, differences between the TDM and non-TDM groups were quantified using RR for both primary and secondary endpoints. Binary outcomes were analyzed through risk ratios with 95% confidence intervals employing the inverse variance method, whereas continuous outcomes were assessed via mean differences with 95% confidence intervals. Considering the varied methodologies and clinical settings of the studies included in this review, significant methodological and clinical heterogeneity was expected. To thoroughly evaluate the statistical heterogeneity among the studies, we utilized the Chi-squared (X^2^) test, where a p-value below 0.05 was deemed indicative of significant heterogeneity.

### 2.7 Sensitivity analysis and publication bias

The Cochrane Risk of Bias assessment showed that most included studies demonstrated moderate to high methodological quality. Detailed domain-specific evaluations for each study are presented in Supplementary File 2. In addition, visual representations of the risk of bias across the included studies are provided in Supplementary Figures S1, 2, both reflecting the methodological quality and bias distribution of the analyzed literature.

To evaluate robustness, we conducted sensitivity analyses that included leave-one-out re-estimation of pooled effects, re-fitting with the alternative model (fixed vs. random effects), and, where applicable, exclusion of studies at higher risk of bias. Consistency of direction and magnitude across these analyses was taken as evidence of stability (see Supplementary File 3). Publication bias and small-study effects were examined when ≥10 studies contributed to a meta-analysis, using visual inspection of funnel plots and Egger’s regression test; no significant small-study effects were detected (Supplementary File 4). Besides, we also introduced Surface Under the Cumulative Ranking (SUCRA) to evaluate and compare the relative performance of different treatment strategies across multiple clinical outcomes, which is a Bayesian framework-based ranking probability method that quantifies the relative advantage of each program by calculating the cumulative ranking area across different metrics. SUCRA values range from 0 to 100, with higher values indicating better performance of the program in the respective metric. Additionally, details of the analytical assays and techniques used for meropenem concentration determination in each study are provided in Supplementary File 5.

## 3 Results

### 3.1 Search results and characteristics of included studies

**FIGURE 1:**
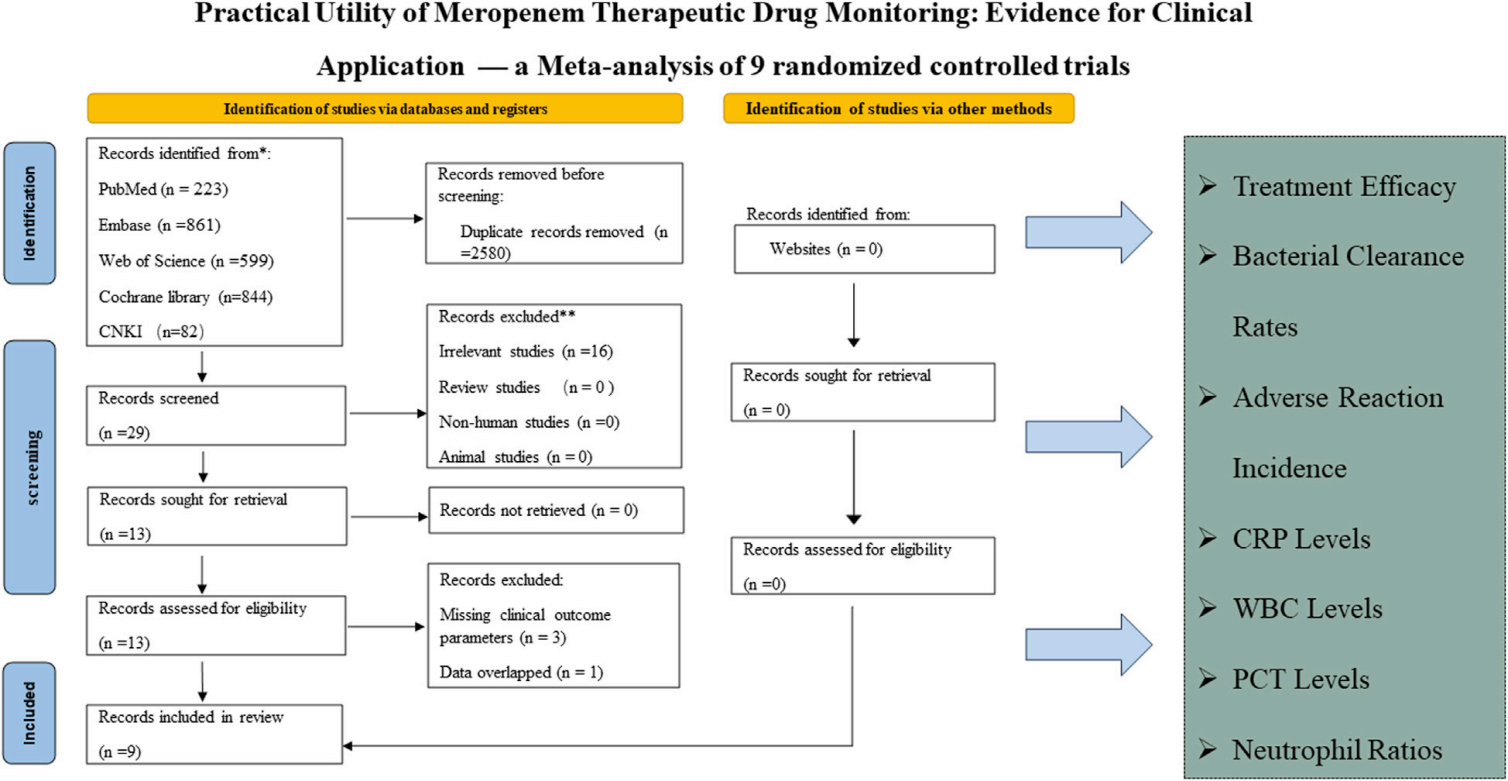
Flow chart demonstrating the inclusion and exclusion of studies into the analysis.

The systematic review commenced with the identification of 2,609 articles across several databases, including PubMed (223 articles), Embase (861), Web of Science (599), Cochrane Library (844), and CNKI (82). The rationale for study selection and exclusion is depicted in Figure 1. Ultimately, nine studies involving a total of 834 patients were included, evenly distributed between TDM and non-TDM groups. The included studies predominantly enrolled adult and elderly cohorts, with reported mean or median ages generally ranging from 50 to 80 years. The infection types varied across studies and chiefly encompassed respiratory tract, urinary tract, and bloodstream infections. The severity of illness was largely moderate to severe, and several investigations included critically ill or ICU patients, underscoring the diverse clinical contexts in which meropenem therapy was employed. The therapeutic regimens differed among studies, with daily meropenem doses ranging from 0.5 g to 3 g and infusion durations varying from 30 min to 24 h, according to the respective study protocols. To enhance methodological rigor, investigators implemented various randomization procedures, including random number tables, stratified sampling, and other standardized allocation techniques. The demographic and clinical characteristics of the included studies are summarized in Table 1, offering a comprehensive depiction of the analyzed populations and fulfilling the reviewer’s recommendation to incorporate detailed patient descriptors.

**TABLE 1:** Details of the included studies.

Study	Treatment regimen		Patients (N)		Male (N)		Age range/Mean ± SD		Daily dose (mg)		Course	Stochastic method
	Exp	Con	Exp	Con	Exp	Con	Exp	Con	Exp	Con		
Lu et al. (2016)	TDM	Con	18	18	NA	NA	55 (42–76)	56 (48–64)	NA	NA	30 min	Treatment method
Bing et al. (2017)	Two step infusion therapy	Con	28	28	20	21	55.9 ± 19.8	61.3 ± 19.2	(0.5–2)g	(0.5–2)g	3 h	Random number table method
Benrun et al. (2023)	TDM	Con	150	150	NA	NA	NA	NA	NA	NA	NA	Stratified sampling method
Yang et al. (2021)	TDM	Con	30	30	18	20	70.27 ± 3.09	70.54 ± 3.23	NA	1 g	8 h	Random number table method
Yu and Aihua (2018)	TDM	Con	56	56	32	31	72.42 ± 13.34	74.84 ± 14.89	NA	NA	NA	Treatment method
Yang et al. (2022)	TDM	Con	33	31	NA	NA	NA	NA	NA	NA	NA	Random
Zhang et al. (2023)	TDM	Con	74	73	51	42	56.26 ± 17.58	61.15 ± 18.31	NA	NA	NA	Treatment method
Zhou et al. (2017)	PK/PD model	Con	39	40	29	24	78 (76–84)	79 (74–87)	NA	NA	NA	Random
Hassanpour et al. (2021)	PK/PD model	Con	9	7	5	4	54.78 ± 24.35	62.43 ± 14.34	3 g (Early phase)/2.17 ± 0.94(Late phase)	3 g	24 h	Random

This table summarizes the key characteristics of the studies included in the systematic review comparing therapeutic drug monitoring (TDM), PK/PD, model–guided therapy, and conventional (control) treatment regimens of meropenem. “Exp” and “Con” denote experimental and control groups, respectively. Treatment regimen: Indicates whether the study applied TDM, PK/PD, model–guided dosing, or conventional administration. Patients (N): Total number of patients enrolled in each group. Male (N): Number of male participants. Age Range/Mean ± SD: Reported patient age distribution or mean ± standard deviation (years). Daily dose (mg): Reported meropenem dosage per day, where available. Course: Duration or frequency of dosing administration (e.g., infusion time or interval). Stochastic method: Method used for patient allocation or randomization, including “random number table method,” “stratified sampling,” or “treatment method” (non-random allocation). Abbreviations: TDM, therapeutic drug monitoring; PK/PD, pharmacokinetic/pharmacodynamic; NA, data not available.

### 3.2 Treatment efficacy

**FIGURE 2:**
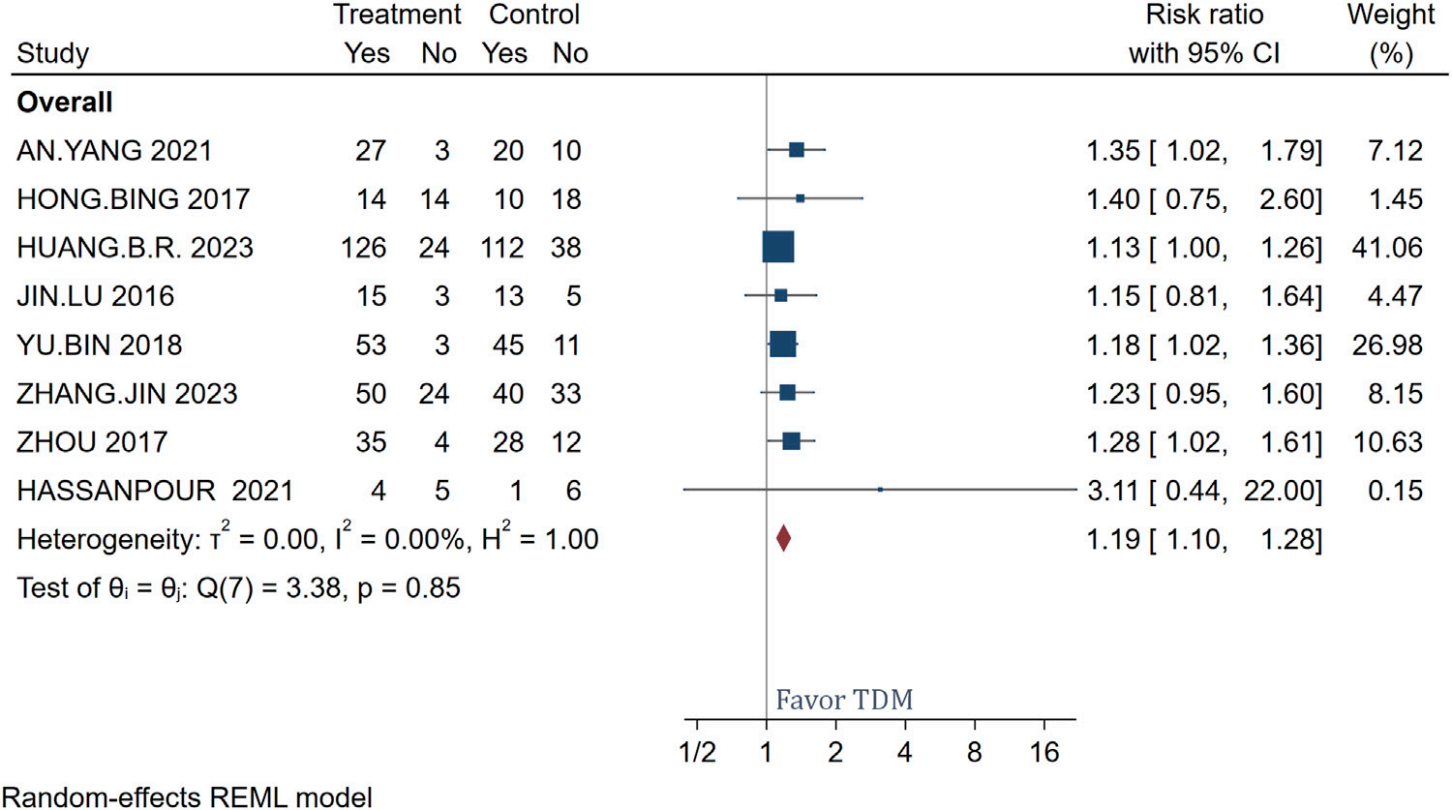
Forest plot of the impact of TDM dosage on treatment efficacy.

This meta-analysis encompassed 604 patients, with 329 in the intervention group receiving meropenem treatment complemented by TDM. As illustrated in Figure 2, the analysis revealed that patients treated with meropenem TDM demonstrated superior therapeutic outcomes compared to the control group of 275 patients who did not receive TDM. This is specifically reflected in a RR of 1.19, with a 95% CI ranging from 1.10 to 1.28, indicating that the TDM treatment group’s effectiveness was 18% higher than that of the non-TDM group. Additionally, heterogeneity tests showed an I^2^ of 0.00% and a p-value of 0.87, indicating a high degree of consistency across the studies with no significant statistical differences, which underscore the importance of drug monitoring in enhancing therapeutic outcomes, supporting the efficacy and consistency of meropenem TDM in clinical applications.

### 3.3 Bacterial clearance rates

**FIGURE 3:**
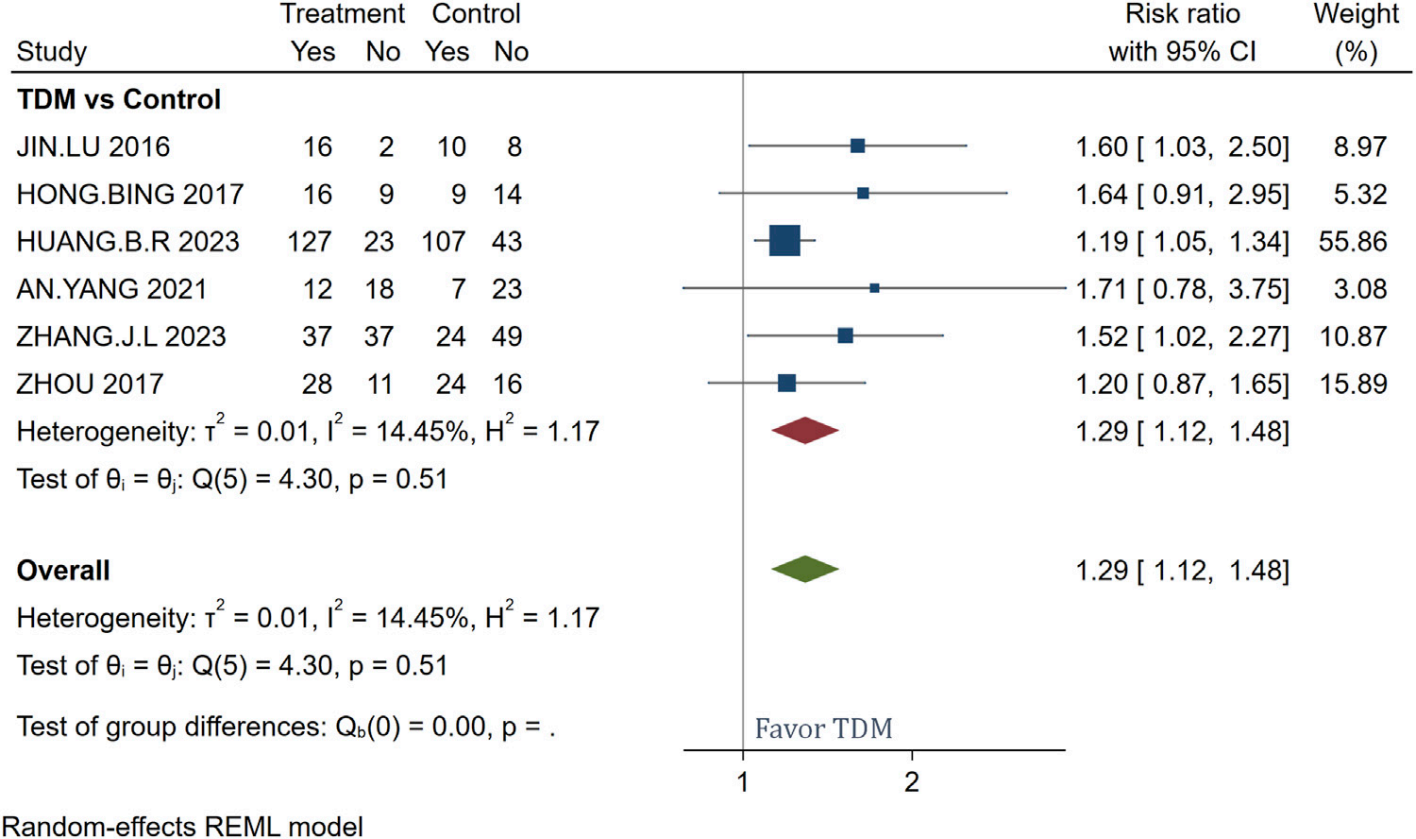
Forest plot of the impact of TDM on bacterial clearance rates.

The meta-analysis of bacterial clearance rates utilized a Random-effects REML (Restricted Maximum Likelihood) model to assess the efficacy of meropenem TDM across six studies involving a total of 417 patients. This model choice is particularly pertinent given the slight variation among studies, as indicated by an I^2^ of 14.45%. The REML model is advantageous for handling heterogeneity in meta-analyses, making it an appropriate statistical approach for combining data from different studies. This approach helped solidify the finding that meropenem TDM increases bacterial clearance rates by 29% compared to non-TDM treatments, as reflected by the overall risk ratio of 1.29 (95% CI [1.12, 1.48]). The consistent results across studies, supported by a p-value of 0.51 (Figure 3), confirm the effectiveness of meropenem TDM in enhancing treatment outcomes.

### 3.4 Adverse effects incidence

**FIGURE 4:**
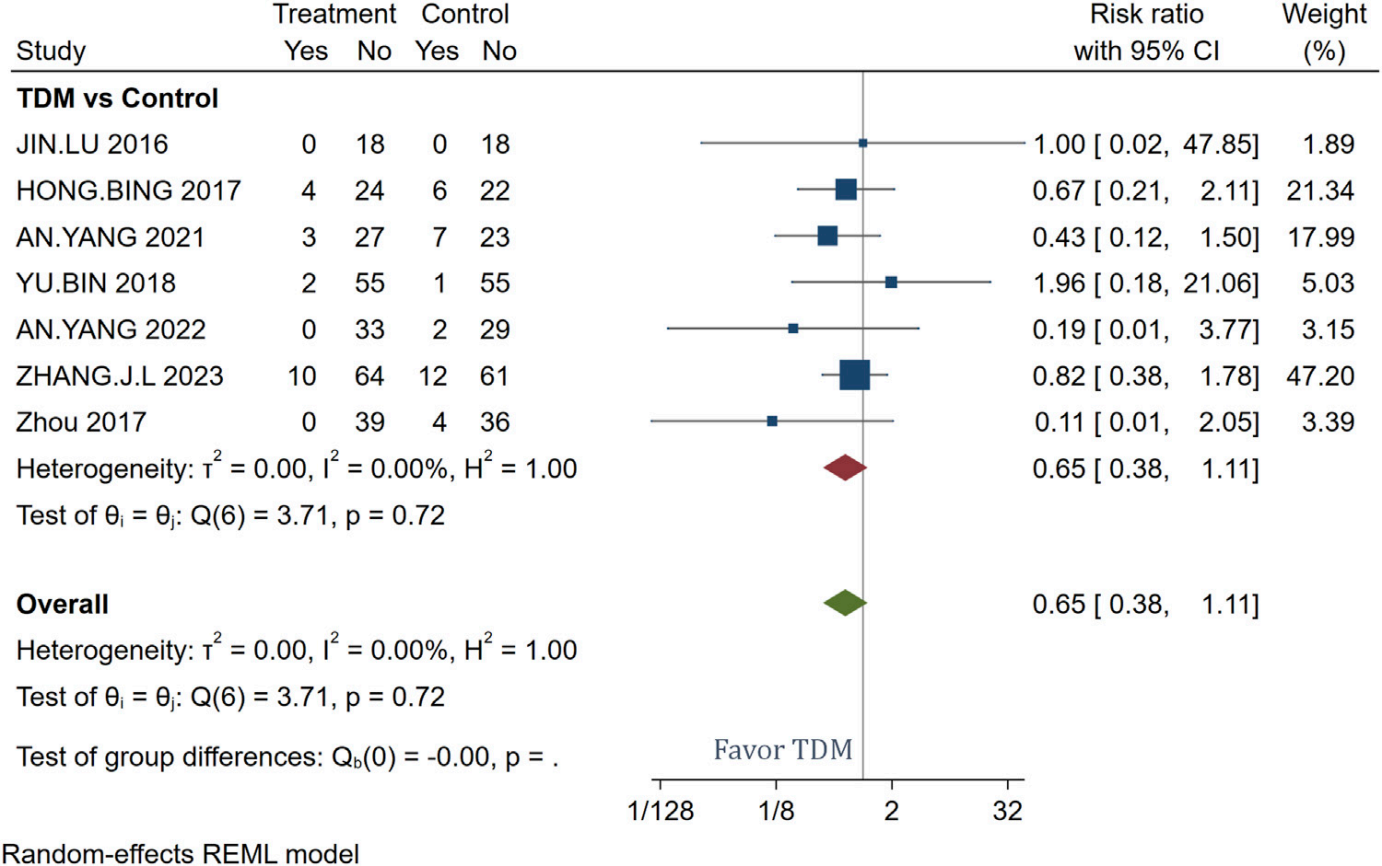
Forest plot of the impact of TDM on adverse reaction incidence.

The analysis includes seven studies that compare the incidence of adverse effects between patients treated with meropenem using TDM and those receiving standard meropenem treatment without TDM. Across these studies, a total of 19 adverse events were reported in the TDM group and 32 in the non-TDM group. The combined risk ratio from the meta-analysis was 0.65 with a 95% confidence interval of [0.38, 1.11], indicating a non-statistically significant trend towards fewer adverse effects in the TDM group, though the confidence interval suggests this result should be interpreted with caution. As summarized in Supplementary File 6, the reported adverse events were predominantly mild and reversible, including renal dysfunction, gastrointestinal reactions, hematologic abnormalities, and mild skin rash. No severe or fatal events were reported in either group. Individual study risk ratios varied widely, from as low as 0.11 to as high as 1.96 (Figure 4), reflecting substantial variability in the individual study outcomes. Despite this variability, the overall heterogeneity among the studies was remarkably low (I^2^ = 0.00%, *p* = 0.72) for the test of overall effect, confirming no significant differences in adverse effects between the groups across the studies.

### 3.5 CRP change

**FIGURE 5:**
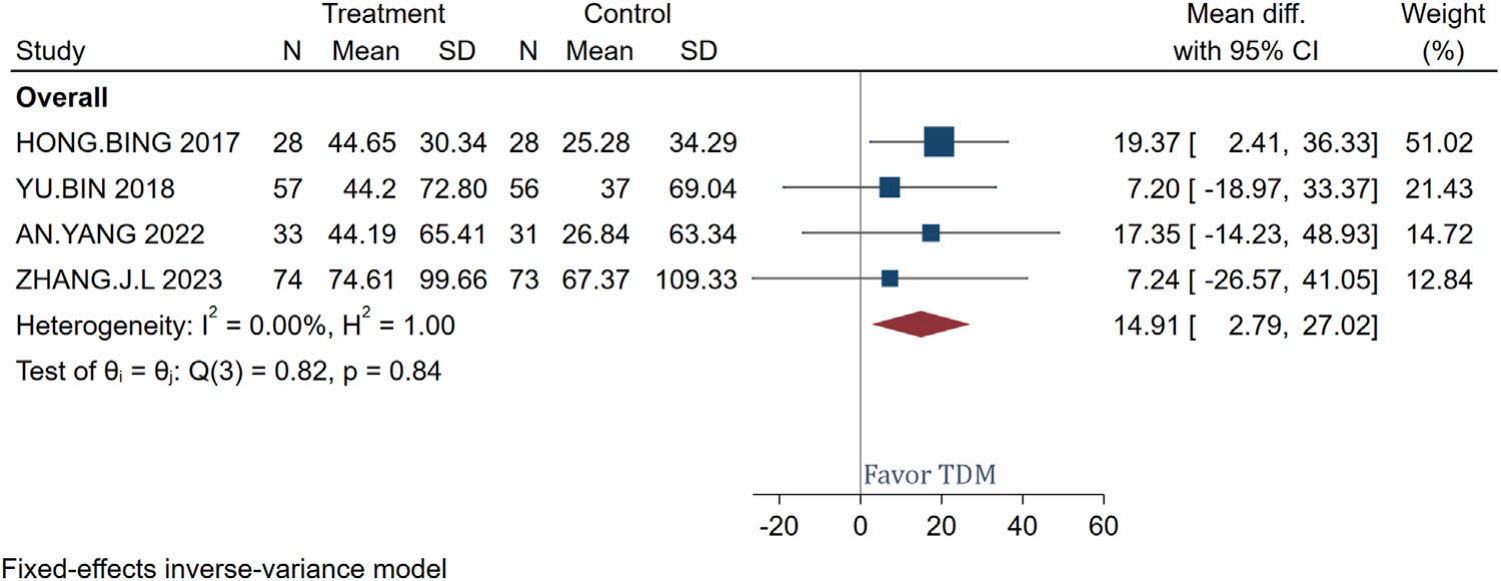
Forest plot of the impact of TDM on CRP Change.

Figure 5 presents the mean differences in CRP levels before and after treatment with meropenem, comparing patients monitored with TDM versus those not monitored. A total of 192 patients were analyzed across the studies included. The use of TDM resulted in a significant reduction in CRP levels compared to standard regimen dosing, with a mean difference of 14.91 and a confidence interval of 2.79–27.02. The study by Bing et al. (2017) showed the most significant effect, with a mean difference of 19.37 and a confidence interval of 2.41–36.33. The heterogeneity test showed very low heterogeneity (I^2^ = 0.00%, H^2^ = 1.00), indicating good consistency among the study results. The overall effect test further confirmed this, with a Q-value of 0.82 and a p-value of 0.84, showing no significant statistical heterogeneity, which supports the efficacy of using TDM in meropenem treatment to reduce CRP levels.

### 3.6 PCT change

**FIGURE 6:**
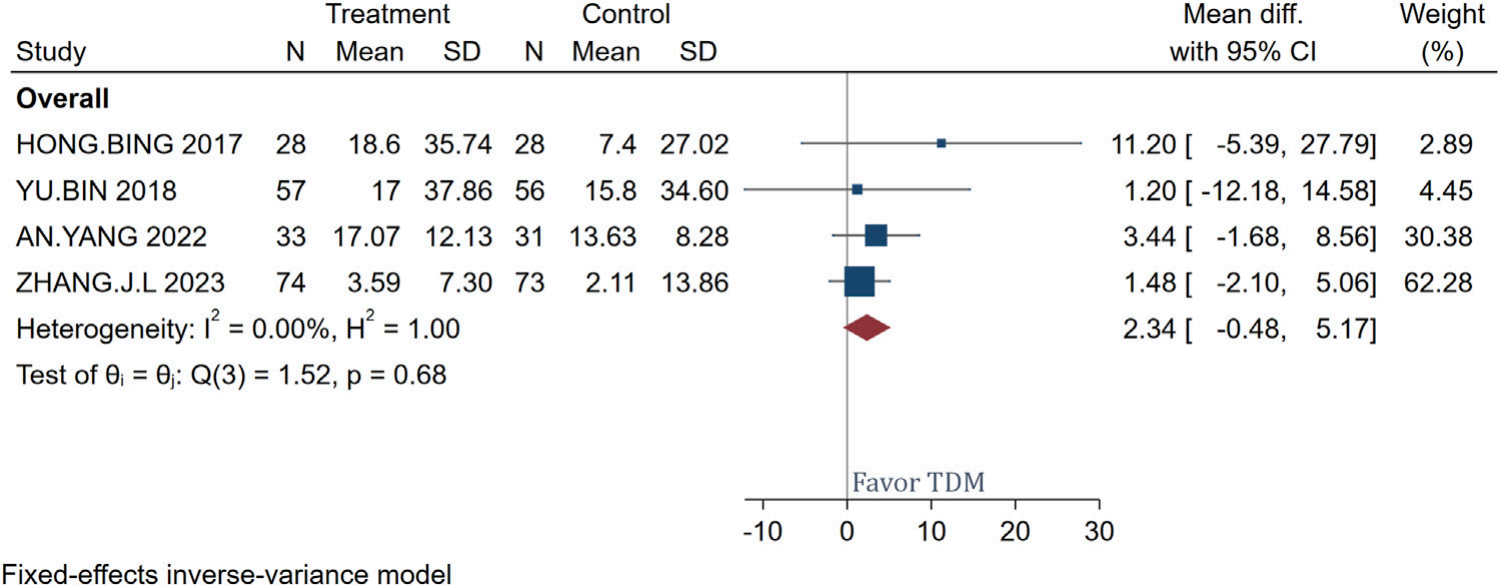
Forest plot of the impact of TDM on PCT change.

Figure 6 presents the mean differences in PCT levels pre- and post-treatment with meropenem, comparing the effects of TDM versus no TDM. The analysis included data from 192 patients. The overall mean difference in PCT levels was calculated to be 2.34, with a confidence interval ranging from −0.48 to 5.17. This demonstrates that, although there were minor fluctuations in PCT levels, these changes lacked statistical significance. Furthermore, the heterogeneity across the included studies was negligible (I^2^ = 0.00%), indicating a high degree of consistency in the findings. The test for overall effect also revealed no significant statistical heterogeneity (Q-value = 1.52, p-value = 0.68).

### 3.7 WBC and neutrophil ratios change

**FIGURE 7:**
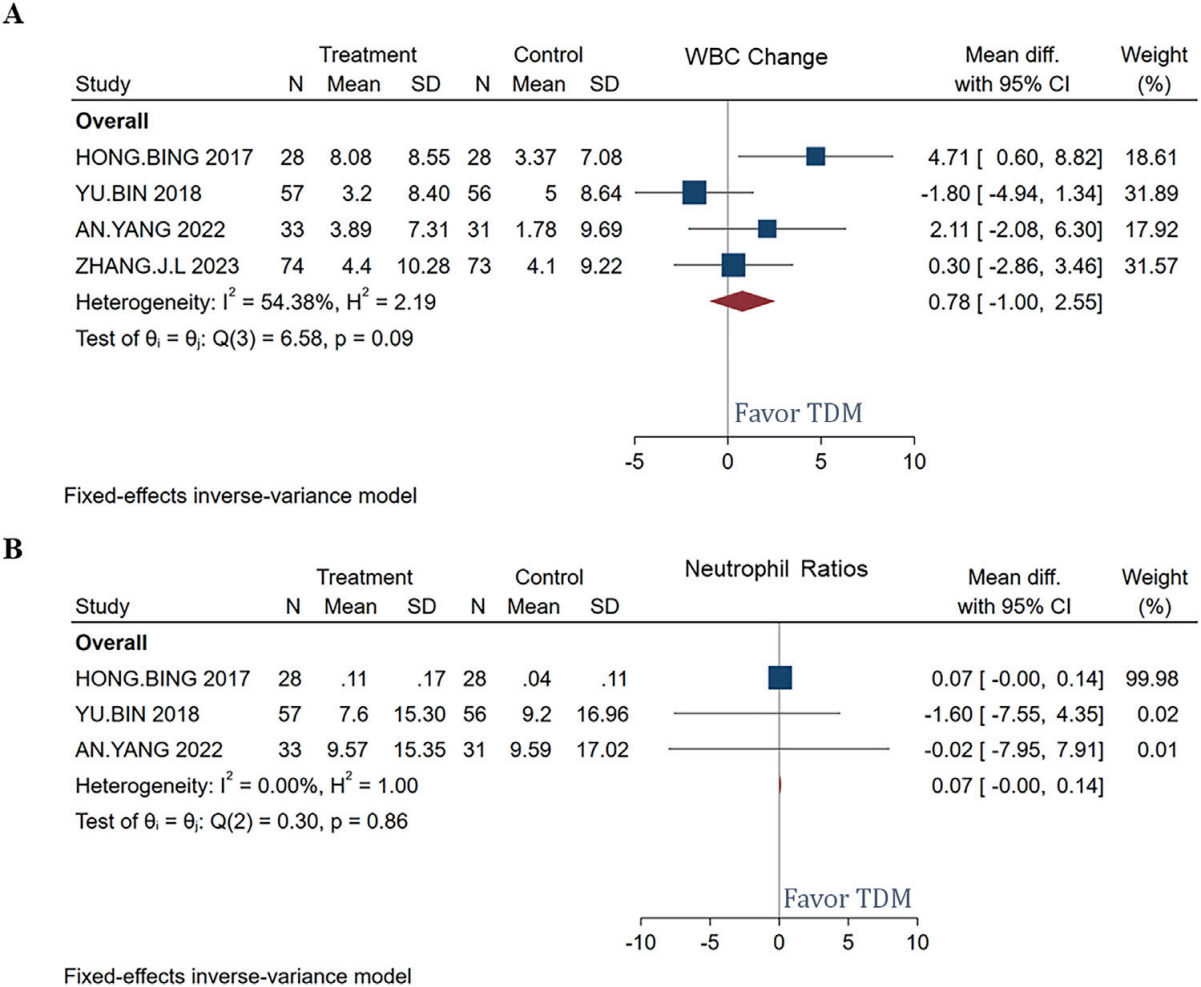
Forest plot of the impact of TDM on WBC **(A)** and neutrophil ratios **(B)** change.

Figures 7A,B respectively display the mean differences in WBC and neutrophil ratios before and after treatment with meropenem, comparing patients managed with TDM to those without TDM management. The analysis included a total of 192 patients. In Figure 7A, the overall mean difference in WBC was 0.78, with a confidence interval ranging from −1.00 to 2.55, indicating that the changes in WBC were not statistically significant. Although the study by Bing et al. (2017) indicated a significant increase, the effects in other studies were not significant. In Figure 7B, the overall mean difference in neutrophil ratios was 0.07, with a confidence interval from −0.00 to 0.14, also showing no statistically significant changes.

### 3.8 SUCRA, bias and sensitivity

Table 2 presents the optimal SUCRA values for each treatment program. The analysis indicates that TDM demonstrates superior performance in terms of changes in CRP levels (SUCRA 99.2), WBC levels (SUCRA 78.3), PCT levels (SUCRA 77.4), neutrophil ratios (SUCRA 96.4), treatment efficacy (SUCRA 100), and bacterial clearance rates (SUCRA 99.2).

**TABLE 2:** The optimal SUCRA values for each treatment program.

Program	CRP levels	WBC levels	PCT levels	Neutrophil ratios	Treatment efficacy	Bacterial clearance rates	Adverse reaction incidence
Treatment	TDM	TDM	TDM	TDM	TDM	TDM	Con
SUCRA	99.2	78.3	77.4	96.4	100	99.2	94.2

## 4 Discussion

There is a growing focus on leveraging TDM to enhance therapeutic effectiveness and minimize drug-related toxicity, particularly for crucial antibiotics like meropenem (Oda et al., 2020; Oda et al., 2021; Restellini and Afif, 2021). However, the majority of existing studies have concentrated on PK/PD targets, such as the percentage of time that the drug concentration remains above the minimum inhibitory concentration (% T > MIC), rather than patient-centered outcomes like clinical efficacy or safety (Casu et al., 2013; Donadello et al., 2015; Scharf et al., 2020a). In practice, TDM is widely applicable to precise antibiotic treatment, especially for meropenem. Studies have shown that in ICU patients, approximately 90% of critically ill patients after liver transplantation can achieve the PK/PD targets when using continuous infusion of β-lactam drugs through TDM (Gatti et al., 2023), while Schmid et al. demonstrated a 10% reduction in meropenem dosing with the use of TDM in ICU patients (Schmid et al., 2023). Beyond ICU patients, TDM is also widely applicable to non-ICU populations: In particular, in the field of urology, TDM provides precise treatment strategies for patients with Gram-negative bacterial infections, effectively balancing the dual objectives of therapeutic efficacy and safety (Berrino et al., 2023). Notably, renal function is a key factor influencing meropenem’s plasma concentrations. For patients with renal dysfunction using meropenem, not necessarily in an ICU setting, TDM can precisely identify patients with abnormal exposure, optimize efficacy and reduce the risk of drug resistance (Angelini et al., 2023). Despite this supporting evidence, and even though current clinical guidelines explicitly recommend TDM for meropenem (Steffens et al., 2021), there is still substantial disagreement regarding the necessity and effectiveness of monitoring (Ong et al., 2007; Pea and Viale, 2009). Consequently, we carried out a systematic review and Meta-analysis to delve into the specific impacts of TDM on the clinical efficacy and safety of meropenem treatment.

In this study, TDM was found to significantly improve clinical efficacy and bacterial clearance rates, consistent with reports emphasizing the contribution of TDM to achieving PK/PD targets (Pea and Viale, 2009; Scharf et al., 2020b; Steffens et al., 2021). The observed reduction in adverse events was limited to a non-significant trend, most likely attributable to the small sample size. Changes in inflammatory markers, including PCT and WBC, did not reach statistical significance, although a trend toward lower PCT levels was observed in patients managed with TDM. The lack of significance may reflect heterogeneity in patient populations or variability in measurement protocols across studies. These findings indicate that, while TDM improves treatment success, its specific influence on inflammatory markers remains inconclusive and requires further systematic evaluation.

Therefore, the findings indicate that the implementation of TDM in the process of meropenem administration can considerably boost clinical efficacy and the rate of bacterial eradication, thus enhancing the overall outcomes of anti-infective treatment. It enables physicians to tailor treatment plans in line with patients’ specific circumstances, apply personalized dosing regimens, and improve the precision of treatment. Moreover, for patients, undergoing TDM during the course of medication can help reduce additional medical expenses that may arise from treatment failure (Conti et al., 2021; Zeng et al., 2021).

Furthermore, the heterogeneity of this study is relatively low. Most trials focused on critically ill or septic patients in Chinese populations, which limits the generalizability of our findings to other settings. Physiological changes unique to ICU patients (e.g., augmented renal clearance) may amplify TDM’s benefits compared to non-critically ill populations (Donadello et al., 2015; Chua et al., 2022). However, this geographic and clinical homogeneity also raises concerns about potential bias, as highlighted in previous meta-analyses. Some studies lacked methodological rigor due to a scarcity of RCTs, limited TDM data, and issues with blinding and reporting (Lechtig-Wasserman et al., 2021). Similarly, a comprehensive review noted that the paucity of randomized trials hindered conclusive findings, despite its extensive scope (Luxton et al., 2022). Therefore, while the low heterogeneity of our study ensures the internal consistency of our results, their external validity remains uncertain, underscoring the need for future studies in diverse geographic and clinical contexts to validate these findings.

Although our study provided positive indications regarding the potential value of TDM in meropenem treatment, due to the limitations of the research design and data conditions, there are still some limitations. Some key limitations temper the interpretation of our results. Initially, the restricted sample size constrains the robustness of statistical inference and the extrapolation of results. Critical outcomes like adverse events relied on small cohorts, reducing the power to detect modest effects. Subsequently, geographical and demographic biases may engender selection bias, thereby impinging upon the external validity of the findings. The overrepresentation of Chinese studies raises concerns about regional practice variations and genetic factors influencing meropenem metabolism. In addition, concomitant pharmacotherapy may introduce confounding. In clinical practice, patients often receive multiple medications, which can influence the outcomes being evaluated. Future research should consider employing more sophisticated statistical methods or study designs that can better account for these potential confounding factors. Moreover, the absence of renal-function data in several studies may have introduced residual confounding. Comprehensive evaluation of kidney function is pivotal for rational antibiotic prescribing, especially when elimination is predominantly renal (Angelini et al., 2025); indeed, deferred renal dose reduction of wide therapeutic index antibiotics could improve outcomes in patients with infectious diseases (Crass et al., 2019). Lastly, heterogeneity in study design similarly exerts an influence on the outcomes: The inclusion of both RCTs and retrospective cohorts may introduce confounding. To illustrate, Machado et al. (2017) reported no mortality benefit with TDM in burn patients, contrasting with our findings in sepsis cohorts, underscoring the impact of patient selection on results. However, this study is still meaningful. It has carried out a relatively comprehensive quantitative analysis of the TDM of meropenem. The results above can also provide a particular reference for clinicians to make a better choice.

In addition, our analysis did not directly assess antimicrobial resistance reduction (due to a lack of data in included studies), TDM’s role in optimizing dosing may theoretically mitigate resistance development by avoiding subtherapeutic exposure. This hypothesis requires validation through dedicated resistance surveillance in future trials. Nevertheless, TDM still shows promise in improving clinical outcomes.

TDM shows promise in improving clinical outcomes, caution is advised in interpreting its effects on adverse events and secondary biomarkers due to limited sample sizes. Future studies with larger cohorts are needed to validate these findings. To ensure the accuracy and rigor of comprehensive analysis conclusions, it is recommended to increase the number of relevant large-sample, multi-center, prospective RCT studies clinically, which would enhance the efficiency and authenticity of research data, providing more scientific evidence for systematically evaluating the efficacy and safety of meropenem TDM. The formulation of age-stratified TDM protocols is also of paramount importance. The pharmacokinetics of meropenem vary in adults and children. In adults, the clearance rate is relatively stable and can be achieved by standard prolonged or continuous infusion to reach the therapeutic target. In contrast, the renal function of newborns and infants is not yet fully mature, and the clearance rate is lower than that of adults, so the dose needs to be adjusted according to the clearance rate (Haseeb et al., 2022). Therefore, it is necessary to develop age-stratified TDM protocols. As an illustration, in neonates, TDM-guided dosing is critical due to rapidly changing pharmacokinetics. De Keukeleire et al. proposed extended infusions (40 mg/kg over 4 h) for optimal exposure (De Keukeleire et al., 2016), whereas Zyryanov et al. emphasized modeling-based approaches for preterm infants (Zyryanov et al., 2023). Additionally, exploring the customized application of TDM for patients with different ethnic backgrounds and baseline disease statuses is crucial. This would help better understand TDM’s actual effectiveness in different healthcare settings worldwide.

In conclusion, TDM-adjusted therapy significantly enhances treatment success and bacterial clearance during meropenem therapy and notably reduces CRP levels; its impact on PCT levels, WBC counts, and neutrophil ratios is not statistically significant. These results may suggest that TDM can optimize certain clinical outcomes but have limited effects on other biomarkers and adverse reactions. Future research should focus on clarifying the clinical utility and potential limitations of TDM in meropenem therapy through well-designed, multicenter RCTs.

## Data Availability

The original contributions presented in the study are included in the article/Supplementary Material, further inquiries can be directed to the corresponding author.

## References

[B1] Abdul-Aziz M. H. Alffenaar J. C. Bassetti M. Bracht H. Dimopoulos G. Marriott D. (2020). Antimicrobial therapeutic drug monitoring in critically ill adult patients: a position paper. Intensive Care Med. 46, 1127–1153. 10.1007/s00134-020-06050-1 32383061 PMC7223855

[B2] Angelini J. Giuliano S. Flammini S. Pagotto A. Lo Re F. Tascini C. (2023). Meropenem PK/PD variability and renal function: we Go together. Pharmaceutics 15, 2238. 10.3390/pharmaceutics15092238 37765207 PMC10534409

[B3] Angelini J. Giuliano S. Lanini S. Ferin S. Martini L. Cossettini S. (2025). In reply to the letter to editor regarding 'renal function and its impact on the concentration of ceftazidime-avibactam: a cross-sectional study. Int. J. Antimicrob. Agents 65, 107480. 10.1016/j.ijantimicag.2025.107480 40057139

[B6] Huang B. R. Xiaocheng L. Hua O. Lingzhao Z. Fan Y. Guocai J. (2023). Exploration and practice of meropenem drug utilization evaluation based on blood concentration monitoring. Chin. J. Hosp. Pharm. 43, 102–106. 10.13286/j.1001-5213.2023.01.17

[B7] Berrino P. M. Gatti M. Rinaldi M. Brunocilla E. Viale P. Pea F. (2023). Pharmacokinetic/pharmacodynamic target attainment of continuous infusion piperacillin-tazobactam or meropenem and microbiological outcome among urologic patients with documented gram-negative infections. Antibiot. (Basel) 12, 1388. 10.3390/antibiotics12091388 37760685 PMC10525318

[B9] Bing H. Lufeng H. Xiaomin Z. Xiuhua Z. (2017). Analysis of the therapeutic effect of the meropenem two-step drip method in treating severe infections in the intensive care unit. Chinese J. Hospital Pharm. 37 (23), 2383–2386.

[B10] Casu G. S. Hites M. Jacobs F. Cotton F. Wolff F. Beumier M. (2013). Can changes in renal function predict variations in β-lactam concentrations in septic patients? Int. J. Antimicrob. Agents 42, 422–428. 10.1016/j.ijantimicag.2013.06.021 23993066

[B11] Chua N. G. Loo L. Hee D. K. H. Lim T. P. Ng T. M. Hoo G. S. R. (2022). Therapeutic drug monitoring of meropenem and piperacillin-tazobactam in the Singapore critically ill population - a prospective, multi-center, observational study (BLAST 1). J. Crit. Care 68, 107–113. 10.1016/j.jcrc.2021.12.013 34999376

[B12] Conti R. M. Padula W. V. Becker R. V. Salamone S. (2021). The cost-effectiveness of therapeutic drug monitoring for the prescription drug-based treatment of chronic myeloid leukemia. J. Manag. Care Spec. Pharm. 27, 1077–1085. 10.18553/jmcp.2021.27.8.1077 34337991 PMC10390998

[B13] Crass R. L. Rodvold K. A. Mueller B. A. Pai M. P. (2019). Renal dosing of antibiotics: are we jumping the Gun? Clin. Infect. Dis. 68, 1596–1602. 10.1093/cid/ciy790 30219824

[B14] De Keukeleire S. Borrey D. Decaluwe W. Reynders M. (2016). Therapeutic drug monitoring of Meropenem in neonate with necrotizing enterocolitis: a challenge. Case Rep. Infect. Dis. 2016, 6207487. 10.1155/2016/6207487 27703820 PMC5040777

[B15] Donadello K. Antonucci E. Cristallini S. Roberts J. A. Beumier M. Scolletta S. (2015). β-Lactam pharmacokinetics during extracorporeal membrane oxygenation therapy: a case-control study. Int. J. Antimicrob. Agents 45, 278–282. 10.1016/j.ijantimicag.2014.11.005 25542059

[B16] Dräger S. Von Rotz M. Labhardt N. D. Siegemund M. Rentsch K. M. Osthoff M. (2023). Early target attainment with continuous infusion meropenem and piperacillin/tazobactam and utilization of therapeutic drug monitoring in critically ill patients: a retrospective cohort study from 2017 to 2020. Open Forum Infect. Dis. 10, ofad143. 10.1093/ofid/ofad143 37077503 PMC10109529

[B17] Gatti M. Rinaldi M. Laici C. Siniscalchi A. Viale P. Pea F. (2023). Role of a real-time TDM-based expert clinical pharmacological advice program in optimizing the early pharmacokinetic/pharmacodynamic target attainment of continuous infusion beta-lactams among orthotopic liver transplant recipients with documented or suspected gram-negative infections. Antibiot. (Basel) 12, 1599. 10.3390/antibiotics12111599 37998801 PMC10668725

[B18] Gonçalves-Pereira J. Póvoa P. (2011). Antibiotics in critically ill patients: a systematic review of the pharmacokinetics of β-lactams. Crit. Care 15, R206. 10.1186/cc10441 21914174 PMC3334750

[B19] Guilhaumou R. Benaboud S. Bennis Y. Dahyot-Fizelier C. Dailly E. Gandia P. (2019). Optimization of the treatment with beta-lactam antibiotics in critically ill patients-guidelines from the French Society of Pharmacology and Therapeutics (Société Française de Pharmacologie et Thérapeutique-SFPT) and the French Society of Anaesthesia and Intensive Care Medicine (Société Française d'Anesthésie et Réanimation-SFAR). Crit. Care 23, 104. 10.1186/s13054-019-2378-9 30925922 PMC6441232

[B20] Gutiérrez-Gutiérrez B. Salamanca E. De Cueto M. Hsueh P. R. Viale P. Paño-Pardo J. R. (2017). Effect of appropriate combination therapy on mortality of patients with bloodstream infections due to carbapenemase-producing enterobacteriaceae (INCREMENT): a retrospective cohort study. Lancet Infect. Dis. 17, 726–734. 10.1016/S1473-3099(17)30228-1 28442293

[B21] Haseeb A. Faidah H. S. Alghamdi S. Alotaibi A. F. Elrggal M. E. Mahrous A. J. (2022). Dose optimization of β-lactams antibiotics in pediatrics and adults: a systematic review. Front. Pharmacol. 13, 964005. 10.3389/fphar.2022.964005 36210807 PMC9532942

[B22] Hassanpour R. Ziaie S. Kobarfard F. Kouchek M. Miri M. Ahmadi Koomleh A. (2021). Evaluation of pharmacokinetic and pharmacodynamic parameters of meropenem in critically ill patients with acute kidney disease. Eur. J. Clin. Pharmacol. 77, 831–840. 10.1007/s00228-020-03062-0 33409684 PMC7787627

[B23] Higgins J. P. Altman D. G. Gøtzsche P. C. Jüni P. Moher D. Oxman A. D. (2011). The cochrane Collaboration's tool for assessing risk of bias in randomised trials. Bmj 343, d5928. 10.1136/bmj.d5928 22008217 PMC3196245

[B25] Kühn D. Metz C. Seiler F. Wehrfritz H. Roth S. Alqudrah M. (2020). Antibiotic therapeutic drug monitoring in intensive care patients treated with different modalities of extracorporeal membrane oxygenation (ECMO) and renal replacement therapy: a prospective, observational single-center study. Crit. Care 24, 664. 10.1186/s13054-020-03397-1 33239110 PMC7689974

[B26] Lechtig-Wasserman S. Liebisch-Rey H. Diaz-Pinilla N. Blanco J. Fuentes-Barreiro Y. V. Bustos R. H. (2021). Carbapenem therapeutic drug monitoring in critically ill adult patients and clinical outcomes: a systematic review with meta-analysis. Antibiot. (Basel) 10, 177. 10.3390/antibiotics10020177 33578672 PMC7916352

[B27] Lu J. Xuemei L. Haixia Z. Weihong G. (2016). Cohort study on drug monitoring of meropenem therapy in intensive care unit patients medicine guide. Beijing, China: Herald of Medicine.

[B28] Luxton T. N. King N. Wälti C. Jeuken L. J. C. Sandoe J. A. T. (2022). A systematic review of the effect of therapeutic drug monitoring on patient health outcomes during treatment with carbapenems. Antibiot. (Basel) 11, 1311. 10.3390/antibiotics11101311 36289971 PMC9598625

[B29] Machado A. S. Oliveira M. S. Sanches C. Silva Junior C. V. D. Gomez D. S. Gemperli R. (2017). Clinical outcome and antimicrobial therapeutic drug monitoring for the treatment of infections in acute burn patients. Clin. Ther. 39, 1649–1657.e3. 10.1016/j.clinthera.2017.06.008 28705450

[B30] Mortensen J. S. Jensen B. P. Doogue M. (2022). Preanalytical stability of flucloxacillin, piperacillin, tazobactam, meropenem, cefalexin, cefazolin, and ceftazidime in therapeutic drug monitoring: a structured review. Ther. Drug Monit. 44, 709–719. 10.1097/FTD.0000000000000975 35175248

[B31] Muller A. E. Huttner B. Huttner A. (2018). Therapeutic drug monitoring of beta-lactams and other antibiotics in the intensive care unit: which agents, which patients and which infections? Drugs 78, 439–451. 10.1007/s40265-018-0880-z 29476349

[B32] Oda K. Jono H. Nosaka K. Saito H. (2020). Reduced nephrotoxicity with vancomycin therapeutic drug monitoring guided by area under the concentration-time curve against a trough 15-20 μg/mL concentration. Int. J. Antimicrob. Agents 56, 106109. 10.1016/j.ijantimicag.2020.106109 32721597

[B33] Oda K. Fujii S. Yamamoto T. Mayumi T. Takesue Y. (2021). Evaluation of once-daily dosing and target concentrations in therapeutic drug monitoring for arbekacin: a meta-analysis. J. Infect. Chemother. 27, 26–31. 10.1016/j.jiac.2020.08.002 32828677

[B34] Ong C. T. Tessier P. R. Li C. Nightingale C. H. Nicolau D. P. (2007). Comparative *in vivo* efficacy of meropenem, imipenem, and cefepime against *Pseudomonas aeruginosa* expressing mexa-mexb-oprm efflux pumps. Diagn Microbiol. Infect. Dis. 57, 153–161. 10.1016/j.diagmicrobio.2006.06.014 16930925

[B35] Pea F. Viale P. (2009). Bench-to-bedside review: appropriate antibiotic therapy in severe sepsis and septic shock--does the dose matter? Crit. Care 13, 214. 10.1186/cc7774 19519961 PMC2717408

[B36] Restellini S. Afif W. (2021). Update on TDM (therapeutic drug monitoring) with ustekinumab, vedolizumab and tofacitinib in inflammatory bowel disease. J. Clin. Med. 10, 1242. 10.3390/jcm10061242 33802816 PMC8002563

[B37] Scharf C. Liebchen U. Paal M. Taubert M. Vogeser M. Irlbeck M. (2020a). The higher the better? Defining the optimal beta-lactam target for critically ill patients to reach infection resolution and improve outcome. J. Intensive Care 8, 86. 10.1186/s40560-020-00504-w 33292582 PMC7686672

[B38] Scharf C. Paal M. Schroeder I. Vogeser M. Draenert R. Irlbeck M. (2020b). Therapeutic drug monitoring of Meropenem and Piperacillin in critical illness-experience and recommendations from one year in routine clinical practice. Antibiot. (Basel) 9, 131. 10.3390/antibiotics9030131 32245195 PMC7148485

[B39] Schmid S. Koch C. Zimmermann K. Buttenschoen J. Mehrl A. Pavel V. (2023). Interprofessional therapeutic drug monitoring of carbapenems improves ICU care and guideline adherence in acute-on-chronic liver failure. Antibiot. (Basel) 12, 1730. 10.3390/antibiotics12121730 38136763 PMC10740747

[B40] Schoenenberger-Arnaiz J. A. Ahmad-Diaz F. Miralbes-Torner M. Aragones-Eroles A. Cano-Marron M. Palomar-Martinez M. (2020). Usefulness of therapeutic drug monitoring of piperacillin and meropenem in routine clinical practice: a prospective cohort study in critically ill patients. Eur. J. Hosp. Pharm. 27, e30–e35. 10.1136/ejhpharm-2018-001713 32296502 PMC7147564

[B41] Sideri S. Papageorgiou S. N. Eliades T. (2018). Registration in the international prospective register of systematic reviews (PROSPERO) of systematic review protocols was associated with increased review quality. J. Clin. Epidemiol. 100, 103–110. 10.1016/j.jclinepi.2018.01.003 29339215

[B42] Steffens N. A. Zimmermann E. S. Nichelle S. M. Brucker N. (2021). Meropenem use and therapeutic drug monitoring in clinical practice: a literature review. J. Clin. Pharm. Ther. 46, 610–621. 10.1111/jcpt.13369 33533509

[B43] Streit F. Perl T. Schulze M. H. Binder L. (2016). Personalised beta-lactam therapy: basic principles and practical approach. LaboratoriumsMedizin 40, 385–397. 10.1515/labmed-2016-0050

[B44] Tabah A. De Waele J. Lipman J. Zahar J. R. Cotta M. O. Barton G. (2015). The ADMIN-ICU survey: a survey on antimicrobial dosing and monitoring in ICUs. J. Antimicrob. Chemother. 70, 2671–2677. 10.1093/jac/dkv165 26169558

[B45] Wong G. Brinkman A. Benefield R. J. Carlier M. De Waele J. J. El Helali N. (2014). An international, multicentre survey of β-lactam antibiotic therapeutic drug monitoring practice in intensive care units. J. Antimicrob. Chemother. 69, 1416–1423. 10.1093/jac/dkt523 24443514

[B4] Yang A. Chaohui T. Suoyang Z. Lei W. Jianxin X. Xuren Z. (2021). To explore the mechanism of continuous infusion of meropenem in the treatment of elderly patients with sepsis in ICU. J. Pract. Med. 37, 726–729+734. 10.3969/j.issn.1006-5725.2021.06.007

[B5] Yang T. C. Zhang S. Zhang J. Yang Y. Jiang D. Jingjiao L. (2022). To optimize the e.faecalis clinical analysis for the treatment of senile sepsis. Chin. J. Lung Dis. Electron. 15, 661–665. 10.3877/cma.j.issn.1674-6902.2022.05.010

[B8] Yu B. Aihua F. (2018). Influence of drug monitoring meropenem on clinical treatment of sepsis clinical misdiagnosis and mistreatment. Clinical Misdiagnosis and Mistherapy 31, 76–80. 10.3969/j.issn.1002-3429.2018.06.024

[B46] Zeng D. Huang X. Lin S. Lin R. Weng X. Huang P. (2021). Cost-effectiveness analysis of genotype screening and therapeutic drug monitoring in patients with inflammatory bowel disease treated with azathioprine therapy: a Chinese healthcare perspective using real-world data. Ann. Transl. Med. 9, 1138. 10.21037/atm-21-1980 34430579 PMC8350671

[B24] Zhang J. L. Linlin H. Jie H. Hua S. (2023). Therapeutic drug monitoring guide meropenem in the treatment of severe infection clinical efficacy and economic evaluation. Chin. Pharmaceutical Industry 32, 101–105. 10.3969/j.issn.1006-4931.2023.02.024

[B47] Zhao Y. Xiao C. Hou J. Wu J. Xiao Y. Zhang B. (2022). C/MIC > 4: a potential instrument to predict the efficacy of Meropenem. Antibiot. (Basel) 11, 670. 10.3390/antibiotics11050670 35625314 PMC9137711

[B48] Zhou Q. T. He B. Shen N. Liang Y. Sun L. N. (2017). Meropenem dosing based on a population pharmacokinetic-pharmacodynamic model in elderly patients with infection of the lower respiratory tract. Drugs Aging 34, 115–121. 10.1007/s40266-016-0431-9 28097633

[B49] Zyryanov S. Bondareva I. Butranova O. Kazanova A. (2023). Population PK/PD modelling of meropenem in preterm newborns based on therapeutic drug monitoring data. Front. Pharmacol. 14, 1079680. 10.3389/fphar.2023.1079680 37007022 PMC10050386

