# Supplementary material for: Practical utility of meropenem therapeutic drug monitoring: a systematic review of evidence for clinical application
Source: Front Pharmacol. 2025 Dec 11;16:1725419. doi: 10.3389/fphar.2025.1725419 (PMC12736388; doi:10.3389/fphar.2025.1725419)
Supplement: Supplementary file 1 [file Supplementaryfile6.docx]

# Supplementary File 6. Adverse Events Information of Included Studies

| Study (Author, Year) | Sample Size (TDM / non-TDM) | Reported Adverse Events | Type of Adverse Events |
| --- | --- | --- | --- |
| JIN.LU 2016 | 18 / 18 | 0 vs 0 | NA |
| HONG.BING 2017 | 28 / 28 | 4 vs 6 | Rash(1)、Candida albicans(1)、Stenotrophomonas maltophilia(2)/Aspergillus(1),Stenotrophomonas maltophilia(4),Renal dysfunction(1) |
| HUANG.B.R 2023 | 150 / 150 | NA | NA |
| AN.YANG 2021 | 30 / 30 | 3 vs 7 | Gastrointestinal damage(1)、Rash(1)、Hematologic system(1)/Gastrointestinal damage(2)、Rash(2)、Hepatic dysfunction(1)、Hematologic system(2) |
| YU.BIN 2018 | 56 / 56 | 1 vs 2 | NA |
| AN.YANG 2022 | 33 / 31 | 0 vs 2 | NA |
| ZHANG.J.L 2023 | 74 / 73 | 14 vs 24 | Nephrotoxicity(10)、Eosinophilia(4)/Nephrotoxicity(12)、Eosinophilia(12) |
| ZHOU 2017 | 39 / 40 | 0 vs 0 | NA |
| HASSANPOUR 2021 | 9 / 7 | NA | NA |

Summary: Across all included studies, adverse events were primarily mild and reversible, most commonly involving renal dysfunction, gastrointestinal reactions, and mild skin rash. No severe or fatal events were reported in either the TDM or non-TDM groups.
