# Supplementary material for: Practical utility of meropenem therapeutic drug monitoring: a systematic review of evidence for clinical application
Source: Front Pharmacol. 2025 Dec 11;16:1725419. doi: 10.3389/fphar.2025.1725419 (PMC12736388; doi:10.3389/fphar.2025.1725419)
Supplement: Supplementary file 2 [file Supplementaryfile1.docx]

**Supplementary File 1 Search strategy**

**CNKI**

| No. | Query | Results | Date |
| --- | --- | --- | --- |
| #1 | （主题：TDM）OR （主题：治疗药物监测）OR （主题：药物监测）OR （主题：监测）OR （主题：优化） | 1482688 | 06-Mar-24 |
| #2 | （主题：美罗培南） | 3378 | 06-Mar-24 |
| #3 | （主题：RCT）OR （主题：随机对照）OR （主题：临床研究）OR （主题：临床试验）OR （主题：随机）OR （主题：对照） | 4477309 | 06-Mar-24 |
| #4 | #1 AND #2 AND #3 | 82 | 06-Mar-24 |

**PubMed**

| No. | Query | Results | Date |
| --- | --- | --- | --- |
| #1 | Meropenem[Title/Abstract] | 10789 | 06-Mar-24 |
| #2 | Randomized Controlled Trial [Filters] OR Clinical Trial [Filters] | 616892 | 06-Mar-24 |
| #3 | #1 AND #2 | 223 | 06-Mar-24 |

**Embase**

| No. | Query | Results | Date |
| --- | --- | --- | --- |
| #1 | (‘meropenem’/exp OR ‘meropenem’) AND (‘therapeutic drug monitoring’/exp OR ‘clinical trial’) AND [English]/lim AND [Embase]/lim | 861 | 06-Mar-24 |

**The Cochrane library**

| No. | Query | Results | Date |
| --- | --- | --- | --- |
| #1 | Meropenem[Title/Abstract/Keyword] | 847 | 06-Mar-24 |
| #2 | Trials[ Title/Abstract/Keyword] | 2151454 | 06-Mar-24 |
| #3 | #1 AND #2 | 844 | 06-Mar-24 |

**Web of Science**

| No. | Query | Results | Date |
| --- | --- | --- | --- |
| #1 | **TS=**Meropenem | 16561 | 06-Mar-24 |
| #2 | **TS=** Randomized Controlled Trial OR TS= Clinical Trial | 2223322 | 06-Mar-24 |
| #3 | #1 AND #2 | 599 | 06-Mar-24 |
