# Supplementary material for: Practical utility of meropenem therapeutic drug monitoring: a systematic review of evidence for clinical application
Source: Front Pharmacol. 2025 Dec 11;16:1725419. doi: 10.3389/fphar.2025.1725419 (PMC12736388; doi:10.3389/fphar.2025.1725419)
Supplement: Supplementary file 3 [file Supplementaryfile5.docx]

# Supplementary File 5. Analytical assays and methods used for Therapeutic Drug Monitoring (TDM)

This supplementary table summarizes the analytical assays used for meropenem TDM across the included studies, including the analytical platform, detection range, and assay validation details.

| Study (Author, Year) | Analytical Method | Assay Platform / Equipment | Detection Range (µg/mL) | Validation / Notes |
| --- | --- | --- | --- | --- |
| JIN.LU 2016 | HPLC | NA | NA | Hospital laboratory validated method |
| HONG.BING 2017 | NA | NA | NA | NA |
| HUANG.B.R 2023 | NA | NA | 2~40 | NA |
| AN.YANG 2021 | HPLC | Agilent 1260 | NA | Use Monte Carlo simulation |
| YU.BIN 2018 | NA | NA | NA | The initial meropenem dosing was based on patient's Cr-Cl. |
| AN.YANG 2022 | NA | NA | NA | Use Monte Carlo simulation |
| ZHANG.J.L 2023 | HPLC | Shimadzu Nexera X2 LC - 30A and AB Sciex API3200 | NA | Hospital laboratory validated method |
| ZHOU 2017 | NA | NA | NA | NA |
| HASSANPOUR 2021 | HPLC-UV | NA | 2.5–80 | NA |

Abbreviations: HPLC = High-Performance Liquid Chromatography; LC–MS/MS = Liquid Chromatography–Tandem Mass Spectrometry; UV = Ultraviolet detection; CLSI = Clinical and Laboratory Standards Institute; CV = Coefficient of Variation; TDM = Therapeutic Drug Monitoring.
