# Supplementary material for: Practical utility of meropenem therapeutic drug monitoring: a systematic review of evidence for clinical application
Source: Front Pharmacol. 2025 Dec 11;16:1725419. doi: 10.3389/fphar.2025.1725419 (PMC12736388; doi:10.3389/fphar.2025.1725419)
Supplement: Supplementary file 4 [file Supplementaryfile3.docx]

**Sensitivity analysis results**

1. **Treatment Efficacy**

1. **Bacterial Clearance Rates**

1. **Adverse Reaction Incidence**

1. **CRP Change**

1. **PCT Change**

1. **WBC Change**

1. **Neutrophil Ratios Change**
