# Supplementary material for: Practical utility of meropenem therapeutic drug monitoring: a systematic review of evidence for clinical application
Source: Front Pharmacol. 2025 Dec 11;16:1725419. doi: 10.3389/fphar.2025.1725419 (PMC12736388; doi:10.3389/fphar.2025.1725419)
Supplement: Supplementary file 5 [file Image2.pdf]

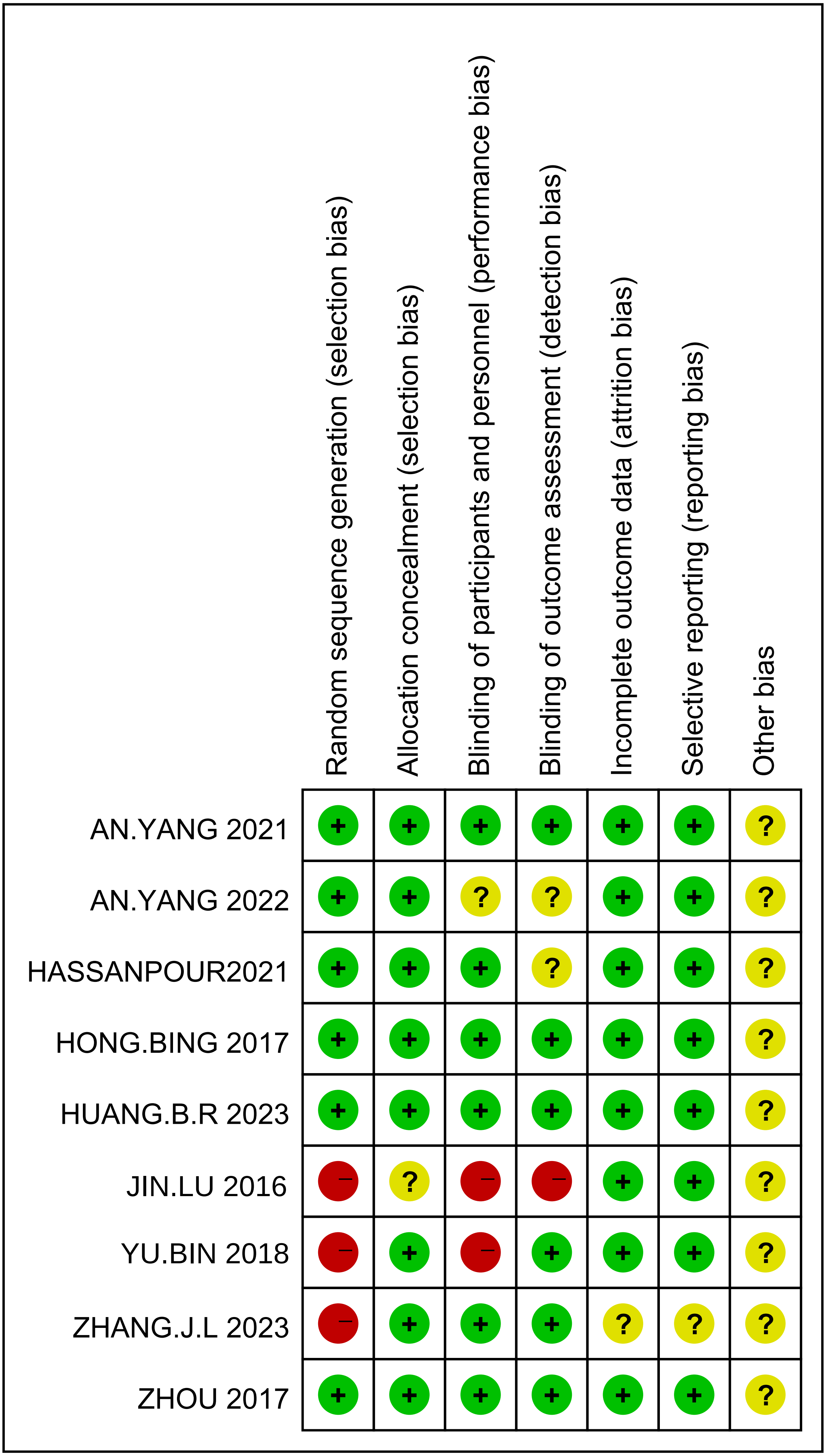

Random sequence generation (selection bias)
Allocation concealment (selection bias)
Blinding of participants and personnel (performance bias)
Blinding of outcome assessment (detection bias)
Incomplete outcome data (attrition bias)
Selective reporting (reporting bias)
Other bias
AN.YANG 2021
AN.YANG 2022
HASSANPOUR2021
HONG.BING 2017
HUANG.B.R 2023
JIN.LU 2016
YU.BIN 2018
ZHANG.J.L 2023
ZHOU 2017
