# Supplementary material for: Practical utility of meropenem therapeutic drug monitoring: a systematic review of evidence for clinical application
Source: Front Pharmacol. 2025 Dec 11;16:1725419. doi: 10.3389/fphar.2025.1725419 (PMC12736388; doi:10.3389/fphar.2025.1725419)
Supplement: Supplementary file 6 [file Supplementaryfile2.docx]

# Supplementary File 2: Risk of Bias Assessment

| Year | Author | Random Sequence Generation (Selection Bias) | Allocation Concealment (Selection Bias) | Blinding of Participants and Personnel (Performance Bias) | Blinding of Outcome Assessment (Detection Bias) | Incomplete Outcome Data (Attrition Bias) | Selective Reporting (Reporting Bias) | Other Bias |
| --- | --- | --- | --- | --- | --- | --- | --- | --- |
| 2016 | JIN.LU | 3 | 2 | 3 | 3 | 1 | 1 | 2 |
| 2017 | HONG.BING | 1 | 1 | 1 | 1 | 1 | 1 | 2 |
| 2023 | HUANG.B.R | 1 | 1 | 1 | 1 | 1 | 1 | 2 |
| 2021 | AN.YANG | 1 | 1 | 1 | 1 | 1 | 1 | 2 |
| 2018 | YU.BIN | 3 | 1 | 3 | 1 | 1 | 1 | 2 |
| 2022 | AN.YANG | 1 | 1 | 2 | 2 | 1 | 1 | 2 |
| 2023 | ZHANG.J.L | 3 | 1 | 1 | 1 | 2 | 2 | 2 |
| 2017 | ZHOU | 1 | 1 | 1 | 1 | 1 | 1 | 2 |
| 2021 | HASSANPOUR | 1 | 1 | 1 | 2 | 1 | 1 | 2 |

Note:

1 = Low Risk: The study demonstrates minimal bias in this domain.
2 = Moderate Risk: Some concerns regarding bias exist, but they are not critical.
3 = High/Unclear Risk: The study either has a high risk of bias or lacks sufficient information to assess bias.
