# Supplementary material for: Practical utility of meropenem therapeutic drug monitoring: a systematic review of evidence for clinical application
Source: Front Pharmacol. 2025 Dec 11;16:1725419. doi: 10.3389/fphar.2025.1725419 (PMC12736388; doi:10.3389/fphar.2025.1725419)

**Supplementary File 4: Egger's test results**

1. **Treatment Efficacy**


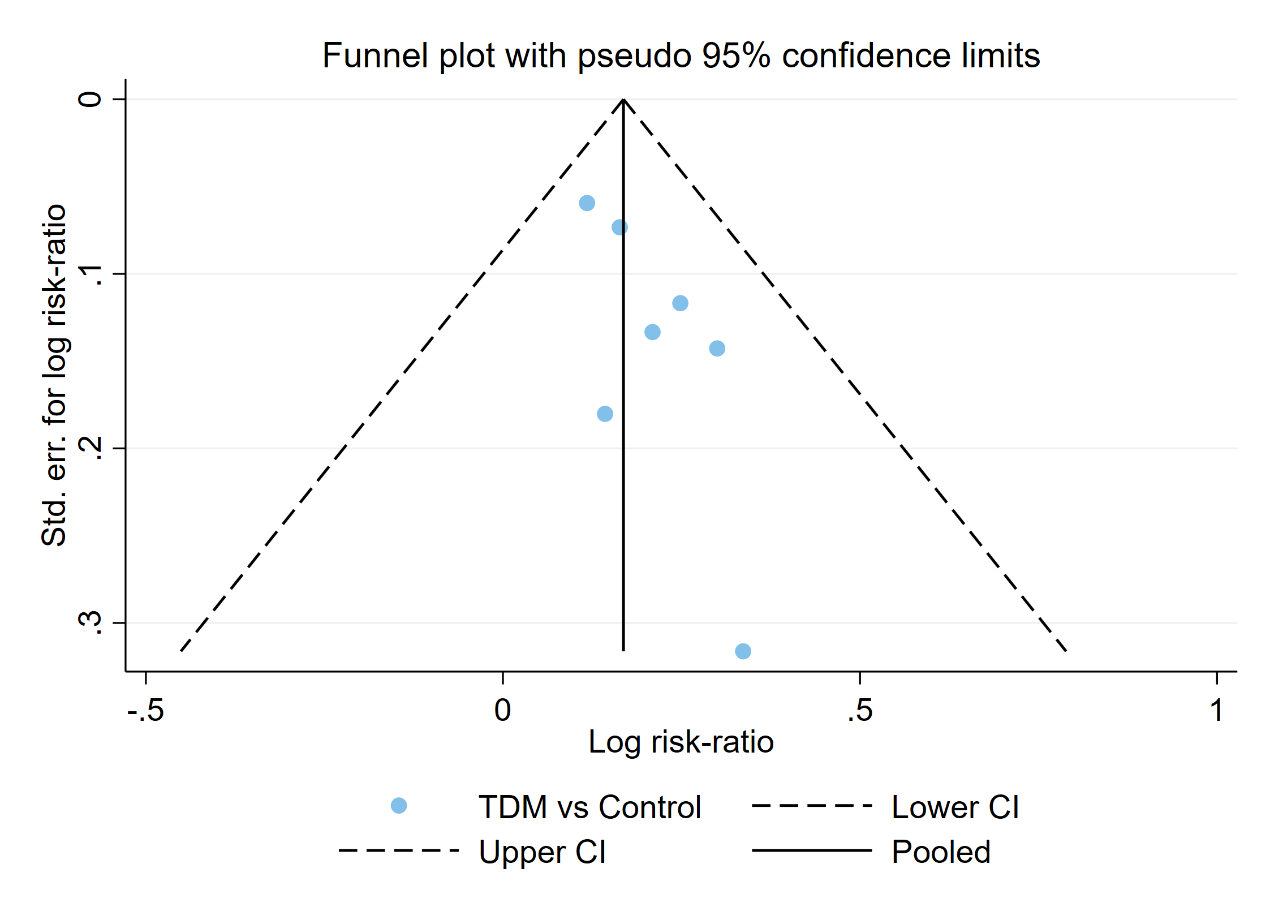


1. **Bacterial Clearance Rates**

**
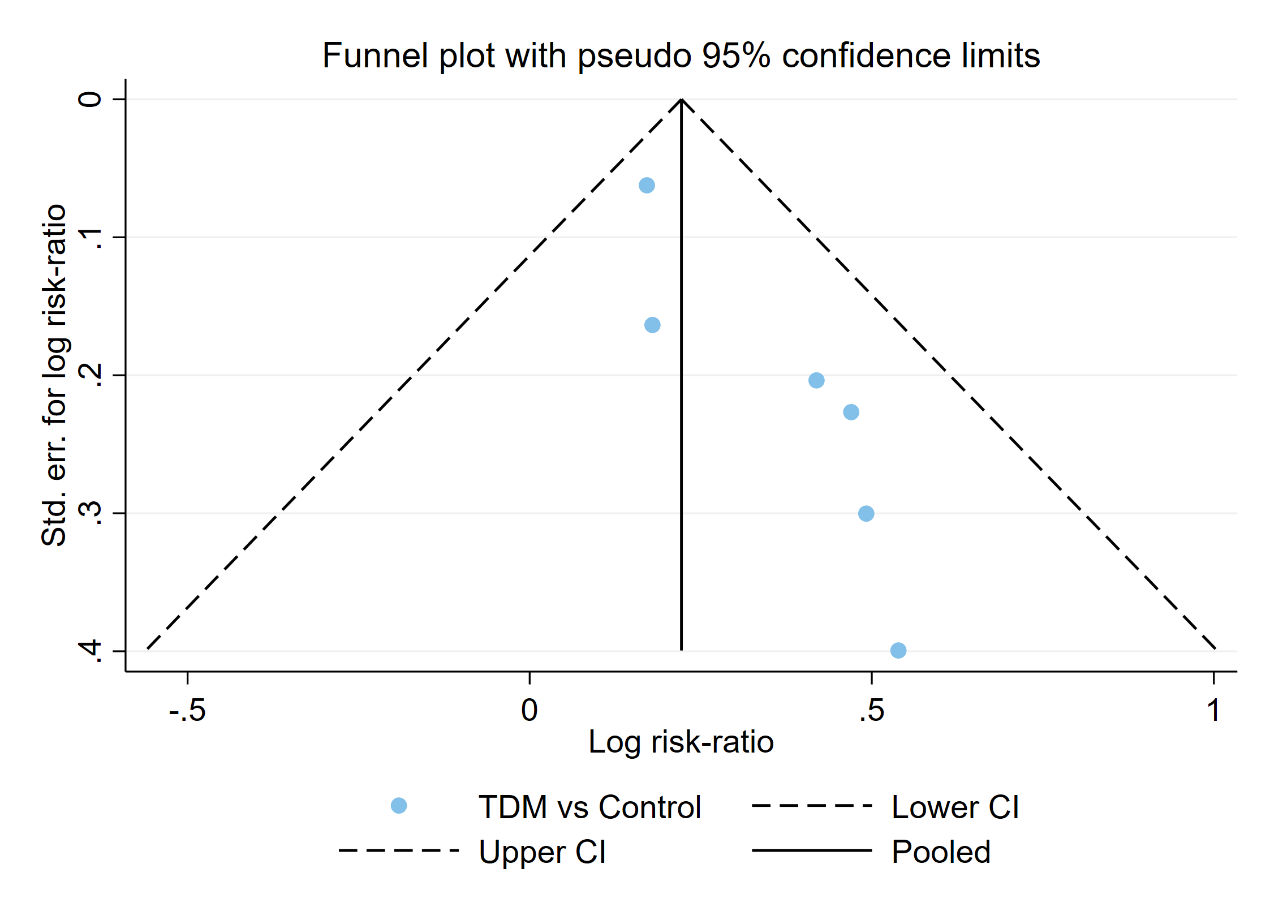
**

1. **Adverse Reaction Incidence**


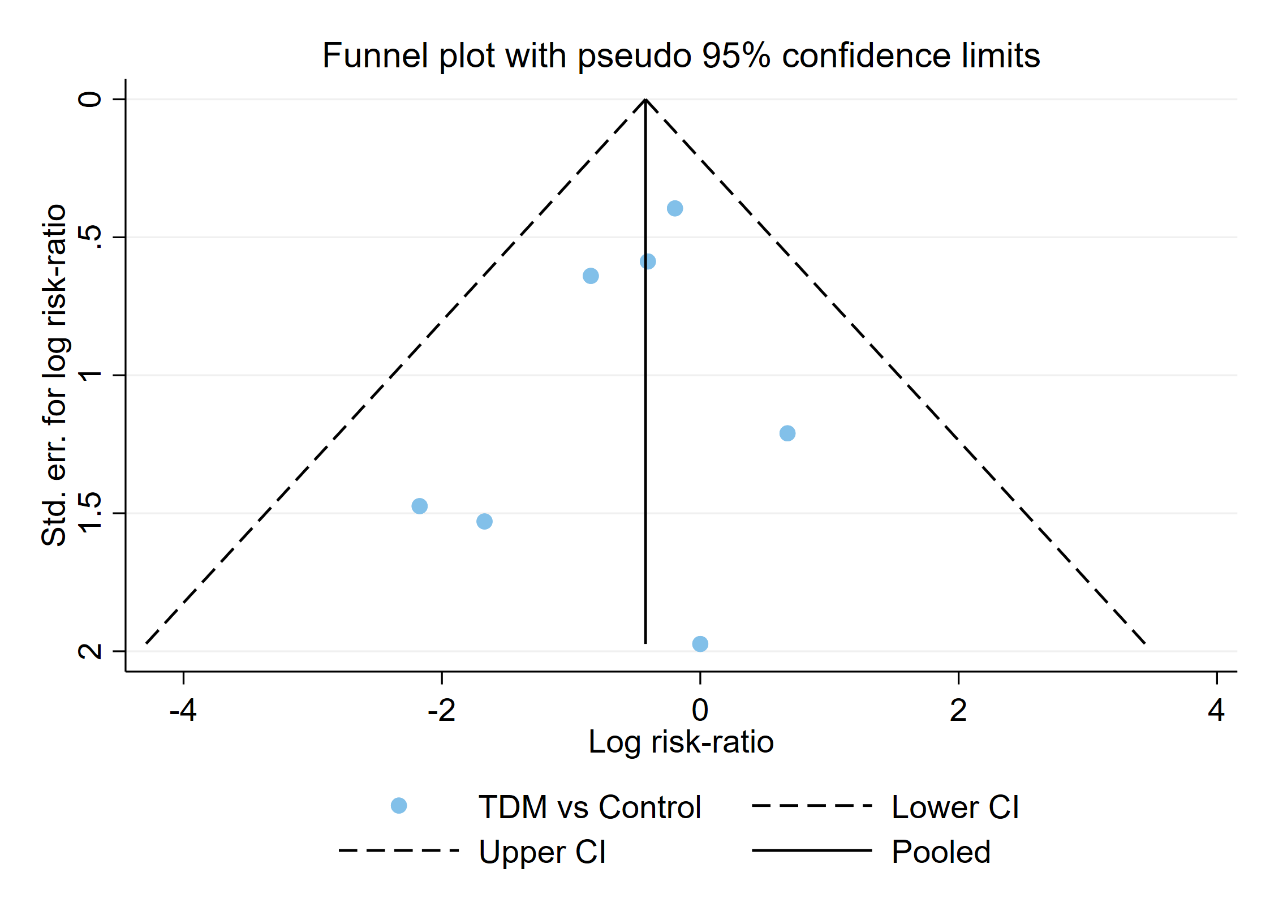


1. **CRP Change**


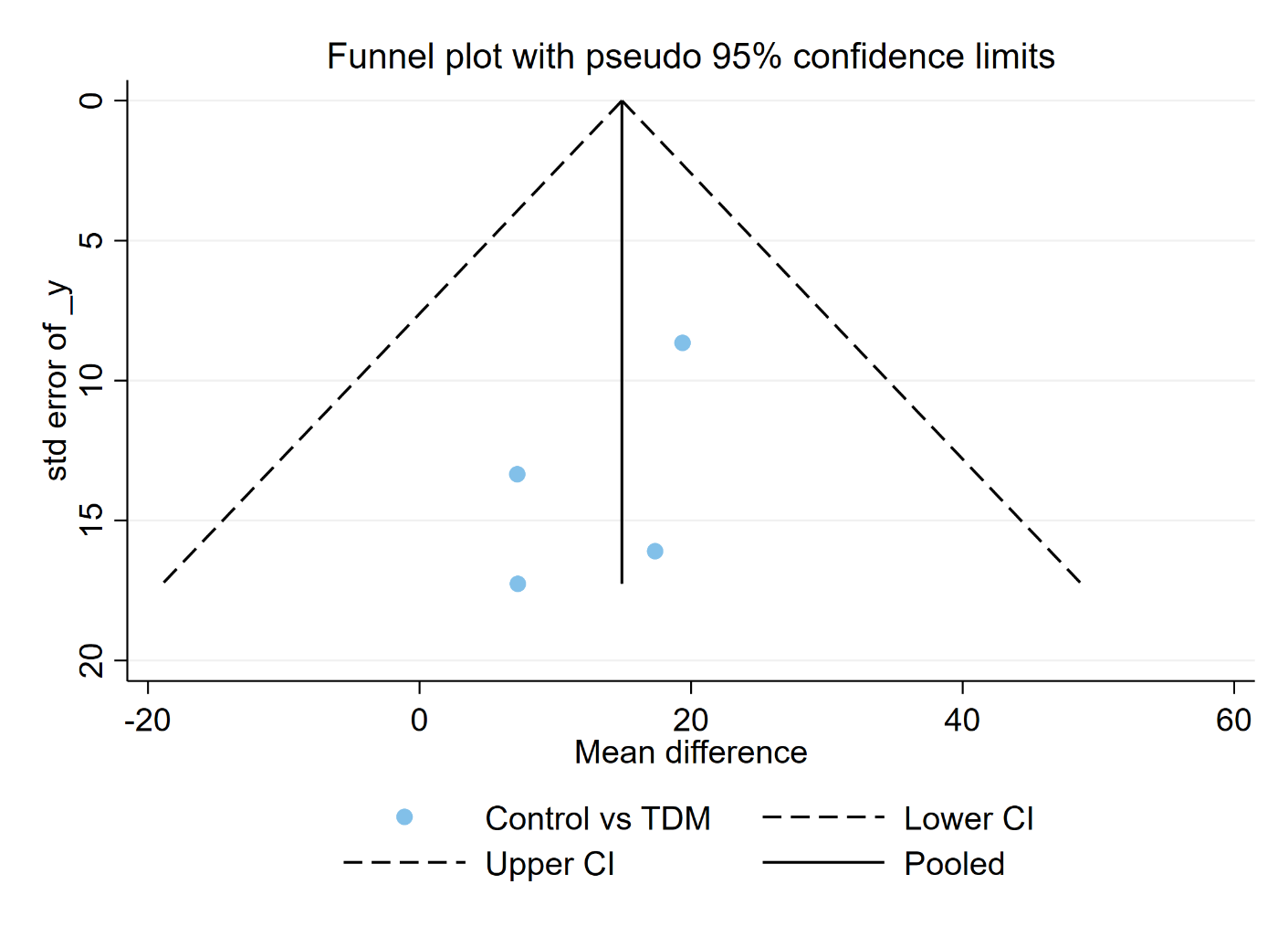


1. **PCT Change**


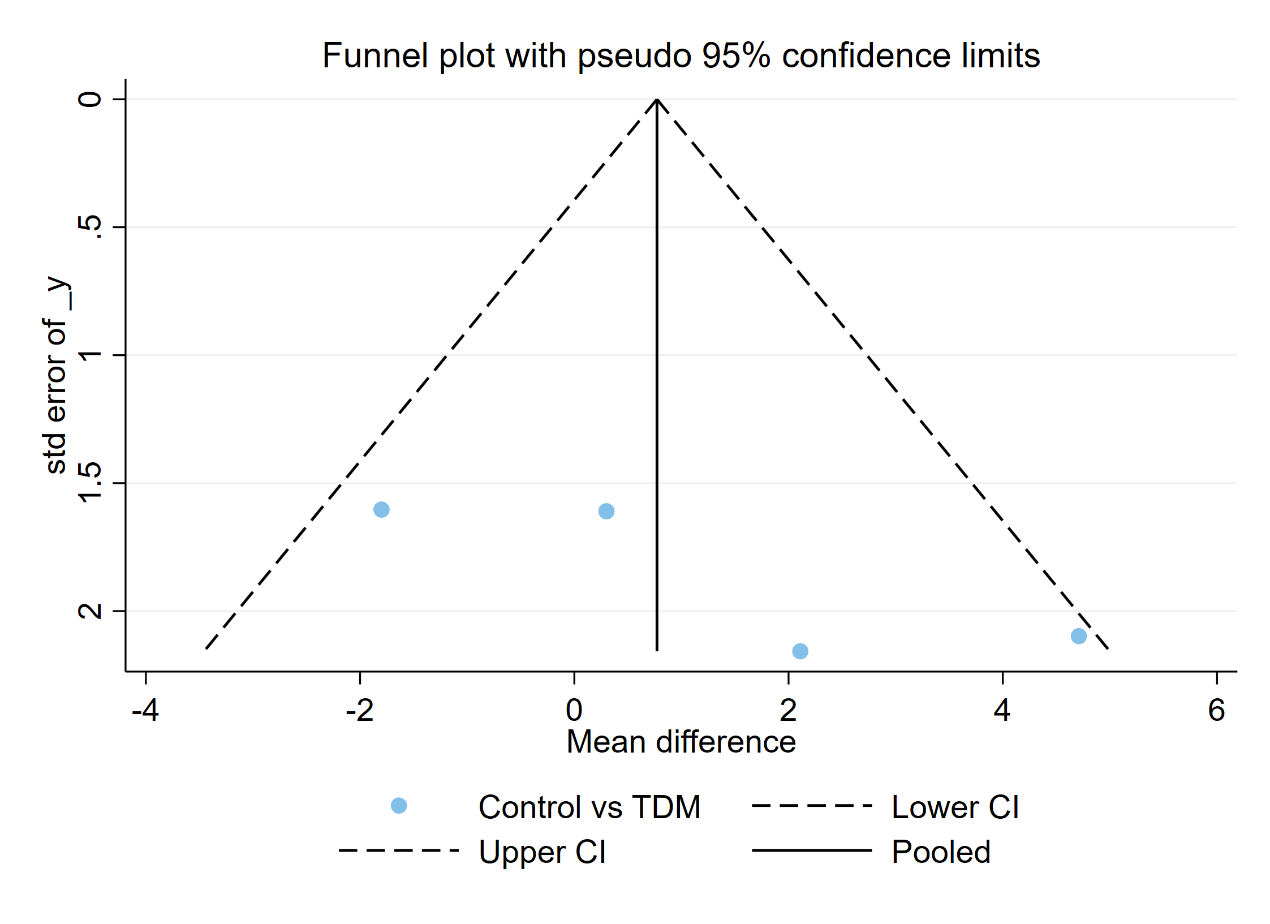


1. **WBC Change**


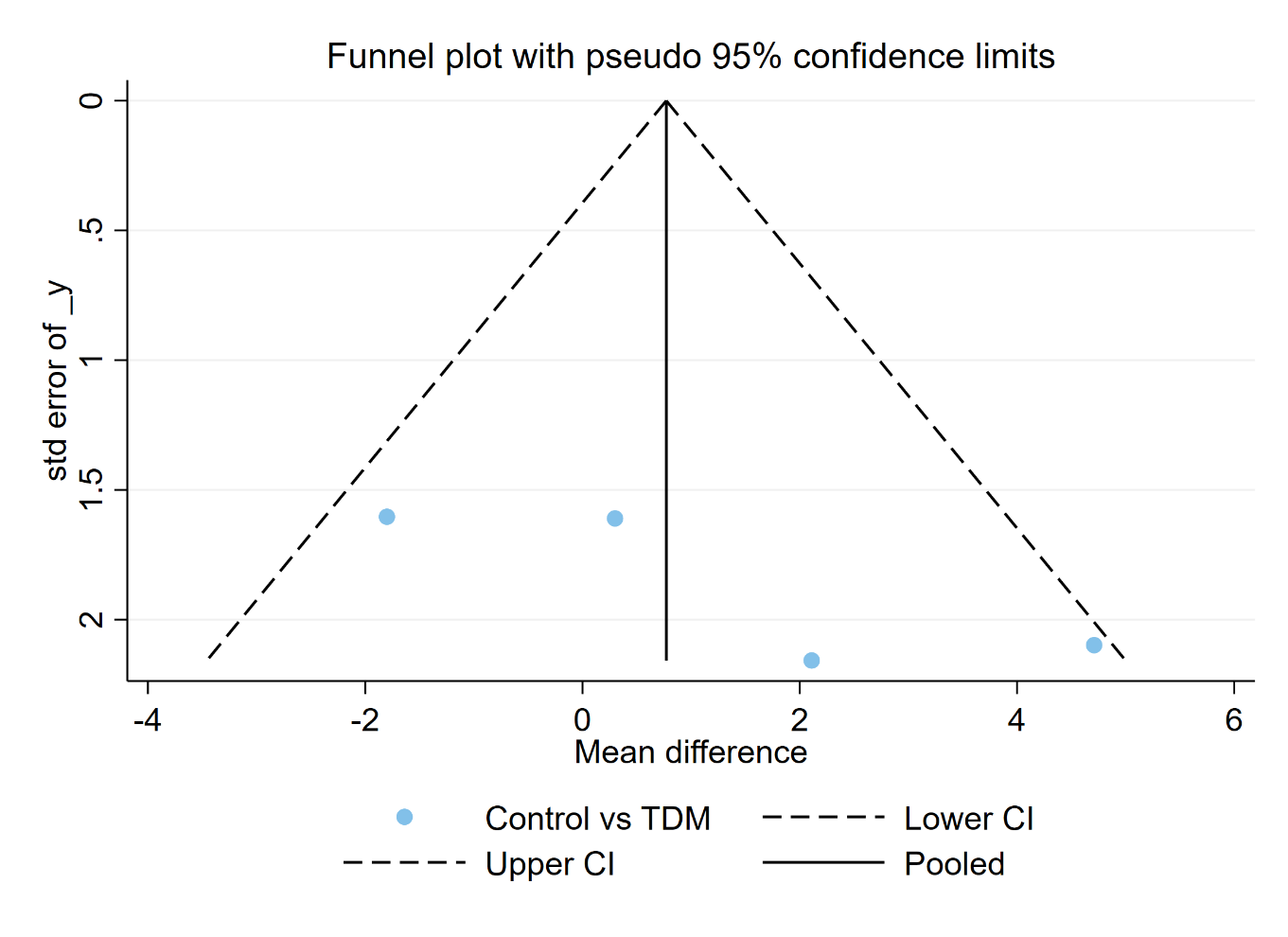


1. **Neutrophil Ratios Change**


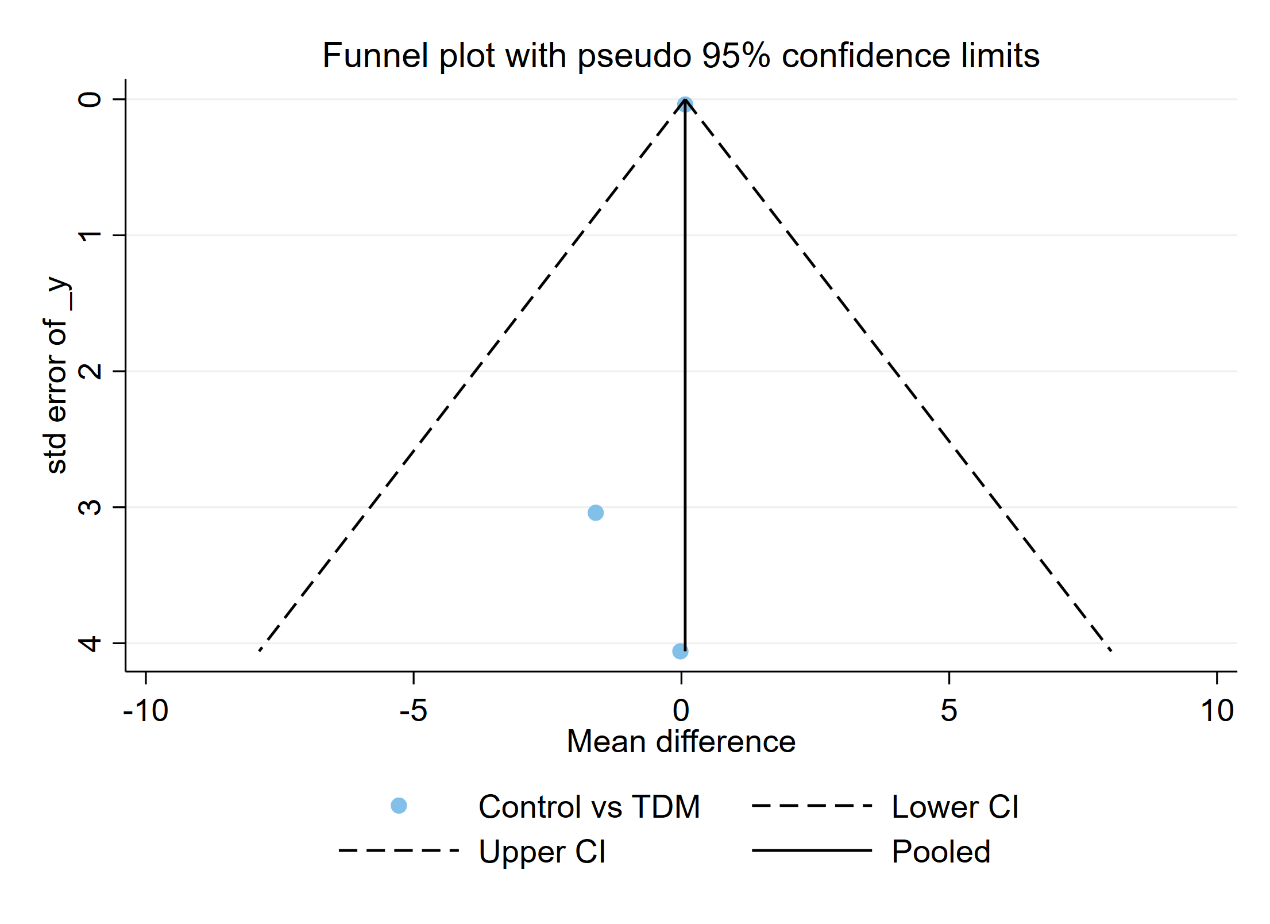

Supplement: Supplementary file 7 [file Supplementaryfile4.docx]
