# Supplementary figures and images for: Practical utility of meropenem therapeutic drug monitoring: a systematic review of evidence for clinical application
Source: Front Pharmacol. 2025 Dec 11;16:1725419. doi: 10.3389/fphar.2025.1725419 (PMC12736388; doi:10.3389/fphar.2025.1725419)

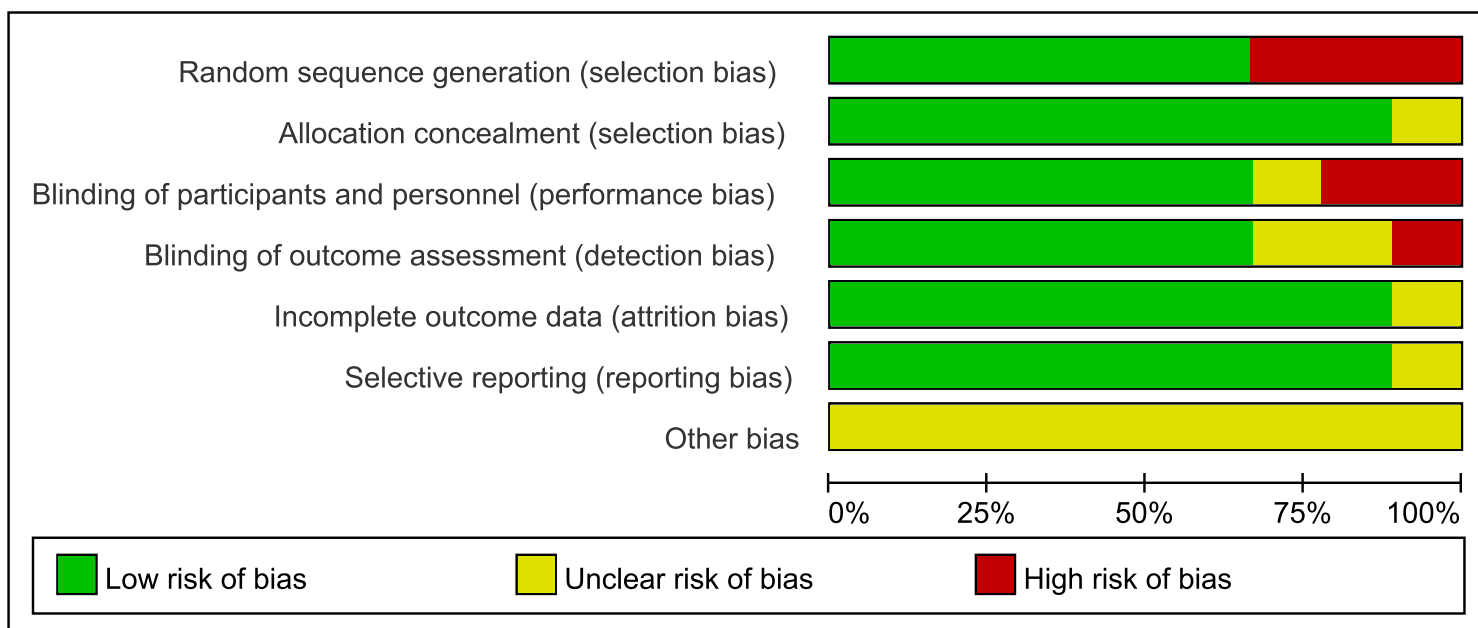

Supplement: Supplementary file 8 [file Image1.pdf]
